# The relationship between physical fitness attributes and sports injury in female, team ball sport players: a systematic review

**DOI:** 10.1186/s40798-020-00264-9

**Published:** 2020-09-14

**Authors:** Jessica B. Farley, Lily M. Barrett, Justin W. L. Keogh, Carl T. Woods, Nikki Milne

**Affiliations:** 1 Faculty of Health Sciences and Medicine, Bond Institute of Health and Sport, Bond University, Gold Coast, QLD Australia; 2 Physical Education and Sport Sciences, University of Limerick, Limerick, Ireland; 3 Sports Performance Research Centre New Zealand, AUT University, Auckland, New Zealand; 4 Cluster for Health Improvement, Faculty of Science, Health, Education and Engineering, University of the Sunshine Coast, Sunshine Coast, Australia; 5 Kasturba Medical College, Mangalore, Manipal Academy of Higher Education, Manipal, Karnataka India; 6 Institute for Health and Sport, Victoria University, Melbourne, VIC Australia

**Keywords:** Women, Injury prevention, Fitness characteristics, Team sports

## Abstract

**Background:**

Understanding the relationships between physical fitness characteristics and sports injury may assist with the development of injury minimisation programs. The purpose of this systematic review was to investigate the association between physical fitness attributes and sports injury in female, team ball sport players.

**Methods:**

Four scientific databases (MEDLINE, EMBASE, SPORTDiscus, Scopus) and reference lists of relevant research were searched for eligible studies up to September 2, 2019. Full-text articles examining the relationship between physical fitness and sports injury in female, team ball sport players were included. A modified Downs and Black checklist was used to assess methodological quality. Data synthesis determined summary conclusions based on the number of significant relationships divided by the total relationships investigated and reported as a percentage. Level of certainty was identified for summary conclusions based on level of evidence. Sub-analyses regarding competition level, age, and single injury types were also conducted.

**Results:**

A total of 44 studies were included. Data synthesis revealed no associations (low to moderate certainty) between body composition (1/9; 11%), flexibility (18–20%), and balance (2/8; 25%) and ‘any injury’ classification. No associations (mostly of moderate certainty) were found between flexibility (0–27%), muscular strength (0–27%), and body composition (14–33%) and various body region injury classifications, whereas mixed summary conclusions were shown for balance (0-48%). Many associations between physical fitness and sports injury were deemed ‘unknown’ or with an insufficient level of certainty. Sub-analyses revealed no association between strength and noncontact ACL injuries (0/5; 0%) or ankle sprains (0/12; 0%), and between flexibility and ankle sprains (1/5; 20%); however, insufficient certainty of these results exists. Clear associations were concluded between balance and lower body injuries in female, non-elite (10/16; 63%) and junior (9/12; 75%) team ball sport players, with moderate and insufficient certainty of these results, respectively.

**Conclusion:**

Limited evidence is available to demonstrate relationships between physical fitness and sports injury in female, team ball sport players. High-quality evidence investigating the multifactorial nature of sports injury, including the interactions physical fitness qualities have with other injury determinants, is needed to better understand the role of physical fitness in minimising sports injuries in female, team ball sport players.

**Trial Registration:**

CRD42017077374 (PROSPERO on September 14, 2017).

## Key Points

- The majority of physical fitness components were not associated, the relationship summary conclusion was deemed unknown, or the summary conclusion identified had an insufficient level of certainty to support the results, regarding sports injury in female, team ball sport players.
- A gap in the literature remains for demonstrating the relationship between physical fitness and injury to support development of injury minimisation programs in female, team ball sport players.
- Future research investigating the multifactorial nature of sports injury is needed to better understand the role of physical fitness and its interactions with other injury determinants in female, team ball sport players, especially considering physical fitness is commonly targeted in injury minimisation studies and programs.

## Background

A sports injury is an inherent risk in sports participation. A detailed understanding of the aetiology of sports injuries is a crucial step in evidence base injury prevention in athletic populations [1]. Models addressing injury mechanisms and aetiology have been published, and have evolved to address this critical sequence in injury prevention research [2–4]. One consistent factor in these models involves the interaction of intrinsic (internal) and extrinsic (external) risk factors [2–4]. Intrinsic risk factors, such as age, sex, flexibility, previous injury, and somatotype, are unique to each athlete, whereas extrinsic risk factors include conditions external to the athlete, consisting of the playing environment, game conditions, and officiating decisions [4]. Given this, it is unsurprising to note the substantial work invested into the understanding of potential intrinsic and extrinsic risk factors likely to associate with sports injury [5–16]. These risk factor categories can be further partitioned into modifiable and non-modifiable risk factors [17]. Whilst understanding both modifiable and non-modifiable risk factors are important for targeted injury minimisation measures, identifying potentially modifiable risk factors may be beneficial for sport practitioners to intervene through specific training programs [18].

The aetiology of sports injuries is multifactorial in nature encompassing a range of factors [3, 17]. Assessment of physical fitness characteristics is one way to identify potential intrinsic, modifiable sports injury risk factors [19]. Physical fitness can be defined as a set of attributes that an individual has or achieves, relating to their ability to perform daily tasks [20]. These physical fitness attributes include the components of agility, balance, body composition, cardiovascular fitness, coordination, flexibility, muscular endurance, muscular strength, power, reaction time, and speed [20, 21]. Previous reviews have aimed to understand potential physical fitness risk factors for sports injury [6, 7, 9–13]. Findings from reviews highlighted some physical fitness injury risk factors, such as decreased hip adductor strength demonstrated a relationship with increased groin injury [12, 13]. Increased quadriceps peak torque was also associated with increased hamstring strain in elite male Australian footballers [7]. Additionally, subsequent research has demonstrated decreased eccentric hamstring strength and between-limb eccentric hamstring strength imbalances were associated with increased risk of hamstring strain injury in elite male Australian footballers [22] and elite rugby union players [23], respectively. In a review by Hrysomallis [9], it was concluded that poor balance was significantly associated with an increased risk of ankle injury during sports participation, with the association more prominent in males than females. Whilst previous reviews have provided insight into potential physical fitness risk factors for sports injuries, some reviews lacked a strength of recommendation taxonomy to summarise the synthesised information [6, 9–12]. Additionally, some reviews included a variety of sporting populations [7, 11], or were inclusive of other populations at risk for injury, such as military recruits and physical education students [9, 10]. Different physical and training characteristics have been described for determining elite field and court players versus elite endurance athletes compared to their non-elite counterparts [24]. Additionally, research has demonstrated rates of injury to be higher in field and court sports, such as soccer and basketball, compared to individual sports, such as swimming and diving, in high school athletes [25]. Therefore, team ball sport players may experience a different relationship between physical fitness and injury than individual athletes. Finally, previous reviews have commonly included both sexes in the study populations, but did not discuss potential sex differences in their injury risk conclusions [6, 7, 9, 11], thereby limiting sex specificity relative to injury risk mitigation.

Given anatomical and physiological differences exist between males and females, research should elaborate on the implications of sex and gender and report results independently in health-related research [26]. For example, a systematic review reported that men had a greater rate of groin injury compared to women playing the same sport at a collegiate level in the United States (US) [27]. Hamstring strains also have been reported at a higher rate in US male collegiate soccer players compared to their female counterparts [28]. In contrast, research demonstrates that female athletes have a substantially greater risk of an anterior cruciate ligament (ACL) injury than male athletes [29–31]. Specifically, reports state that women are 2–8 times more likely than men to sustain an ACL injury, with greater incidence commonly occurring in athletes participating in pivoting sports, such as soccer and volleyball [31]. This research indicates that there are significant differences in common injuries sustained between male and female athletes participating in competitive sport, notably in team ball sports undergoing similar physical demands and stresses. These sex differences in sports injury incidence rates may be explained by the differences in risk profiles proposed for males versus females for some sports injuries, including differences in anatomical, hormonal, or neuromuscular factors [14, 16, 32–34]. Additionally, research has demonstrated performance and anthropometric differences between male and female athletes in various physical fitness components [35–41]. Furthermore, females are underrepresented in sports science research [42, 43], with male dominated research often bolstering sport science practices, such as injury minimisation programs [43]. Given these differences described above, understanding and integrating sex considerations may be integral when developing appropriate injury minimisation programs to ensure optimal athlete performance and promote player safety for female, team ball sport players.

Understanding intrinsic injury risk factors is just one piece of the comprehensive and multifactorial sequence of sports injury aetiology. Identifying those individuals potentially at high-risk of sustaining a sports injury and who may benefit from an injury minimisation program is one way to target modifiable risk factors; however, it is important to first demonstrate a strong association between the risk factor and sports injury [18]. Therefore, the purpose of this systematic review was to identify and critically appraise the available literature to investigate the associations between physical fitness attributes and sports injury in female, team ball sport players. The synthesis of this work may offer team sport practitioners and researchers a basis to develop targeted training interventions that are sex specific and intended to reduce sports injury in these female players.

## Methods

### Registration

In accordance with the Preferred Reporting Items for Systematic Reviews and Meta-analysis (PRISMA) guidelines [44], this systematic review was registered with the International Prospective Register of Systematic Reviews (PROSPERO) on September 24, 2017 (registration number CRD42017077374).

### Data Sources

To avoid duplication of research, PROSPERO was initially searched for ongoing and previously registered reviews. Five scientific databases [MEDLINE (Ovid interface from 1946 to present), EMBASE (from 1947 to present), SPORTDiscus (from 1985 to present), Scopus (from 1970 to present), and ProQuest (from 1937 to present)] were initially planned to be searched for this systematic review. The ProQuest database platform was removed from the final search application based on recommendation from the university faculty librarian, due to technical changes with their search platform and with sufficient coverage of relevant journals in the retained databases. Hence, the remaining four databases were searched for relevant studies up to September 2, 2019. The reference lists of studies included in the review and of previously published systematic reviews of similar topic were also screened for additional relevant articles.

### Search Strategy

An initial MEDLINE literature search strategy was developed by the chief investigator and their university faculty librarian with expertise in systematic review searching. Utilising the PICO (population, intervention, comparison/control, outcome) format [45], the search strategy included medical subject headings (MeSH) and text words related to the following concepts: female (e.g. “women”, “girl”), team ball sport players (P) (e.g. “athlete”, “ball sport”), physical fitness measures (I/C) (e.g. “strength”, “balance”), and sports injury (O) (e.g. “risk”, “injury”). Prior to conducting the search, the final search strategy was revised by four of the five contributing authors (for complete search strategy, please see Online Resource 1). MeSH and text words were searched in all fields using syntax specific to each database. Search results were condensed using ‘English’ and ‘journal article’ filters.

### Eligibility Criteria

Studies were deemed eligible for inclusion using the criteria outlined below:

#### Study Design

Original research studies of observational design, inclusive of prospective and retrospective cohort studies, case-control studies, cross-sectional studies, case series, and case reports were included. Interventional studies that reported a comparison of baseline data between objective measures, or pre- and/or post-values of objective measures were also included. Interventional studies that did not meet these criteria and literature reviews were excluded.

#### Participants

Female players participating in land-oriented, team ball sports categorised as invasion games, net/wall games, and striking/fielding games were included [46]. Examples of eligible sports included (but was not delimited to) are as follows: basketball, volleyball, cricket, baseball, softball, handball, netball, lacrosse, field hockey, and any football code (Australian football, Gaelic football, American football, flag football, soccer, futsal, indoor soccer, rugby union, rugby league, rugby sevens, touch rugby). All competition levels, such as youth, recreational, sub-elite, and elite, were included. Those studies that investigated both male and female populations were included only if the female players’ subset of data were identifiable and reported separately. Studies that examined exclusively males or female players with a physical or mental disability were excluded, as inclusion of these data may provide distinct associations to those of able-bodied players.

#### Physical Fitness Measures

Studies that performed at least one objective measure of physical fitness were included. This comprised any measure that addressed at least one of the following physical fitness components: (i) agility, (ii) balance, (iii) body composition, (iv) cardiovascular fitness, (v) coordination, (vi) flexibility, (vii) muscular endurance, (viii) muscular strength, (ix) power, (x) reaction time, or (xi) speed. Studies were excluded that solely investigated non-physical fitness attributes, such as psychological or behavioural characteristics, pertinent to sport.

#### Sports Injury Outcomes

For the premise of this review, sports injury encompassed any definition of a recordable physical injury, including all-complaints, medical attention, and time loss [47] (Table 1). For observational studies to be included, they must have reported statistical measures describing associations between a physical fitness measure and sports injury. For experimental studies to be included, they must have delineated pre- and/or post-test values for physical fitness and sports injury outcomes and assessed the relationship between these variables. Associations referring to any psychological complaint relevant to sport participation or illnesses were excluded.

**Table 1:** Types of recordable injuries [47]

Injury identifier	Definition
All-complaints	Any physical complaint applicable to sports participation, regardless of its outcome
Medical attention	Physical injuries receiving medical treatment or evaluation from a medical practitioner
Time loss	Physical injuries resulting in an inability to fully participate in training or competition

Injury identifiers defined are about physical injuries only. Reference to ‘illness’ and ‘psychological complaints’ were excluded from this review

#### Other

Articles published in English only were included. Accessible full-text articles published in peer reviewed journals were included in this review. Electronic searches were limited by the date accessible within each respective database. Handsearching was restricted to articles published prior to September 2, 2019 to remain consistent with the electronic searching methods.

#### Data Management

Electronic and handsearch results were exported into an electronic reference management software program, EndNote (version X8, by Thomson Reuters) for reference storage and identification of duplicates. A web-based software platform, Covidence (Covidence online systematic review platform, Veritas Health Innovation Ltd, Melbourne, Australia, www.covidence.org), was utilised to assess the eligibility criteria against retrieved records, as well as to conduct data extraction and methodological quality assessment of studies included in this review. Covidence is recommended by Cochrane to simplify the construction of systematic reviews [48].

### Selection Process

Titles and abstracts of records generated by the search were screened independently by two reviewers (chief and secondary investigator) for relevance applying the eligibility criteria. Full-text manuscripts were acquired for records that appeared to fulfil, or when it was unclear if met, the eligibility criteria. The same two reviewers independently assessed full-text articles for eligibility with reasons for exclusion documented. Any discrepancies were resolved by discussion between the two reviewers to achieve consensus. The reviewers were not blinded to any identifying information of the eligible records throughout the identification, screening, or eligibility processes.

### Data Extraction

One reviewer (chief investigator) extracted data from each included study using Covidence. Extracted data included are as follows: descriptive information of the study population, study design, physical fitness attribute(s) measured, data collection methods, and sports injury outcome utilised. Described sports injury outcomes were then classified by injury identifiers outlined in Table 1. Statistical analyses used and main findings reported regarding the relationship data between physical fitness measure(s) and sports injury were also extracted.

### Critical Appraisal of Methodological Quality in Individual Studies

Two reviewers independently appraised the methodological quality of included studies using a modified Downs and Black protocol [49]. The Downs and Black protocol was developed to assess the methodological quality of randomised controlled trials (RCTs) and non-randomised studies [49]. Given the eligibility criteria for this review included both interventional and observational study designs, the Downs and Black protocol was utilised due to the robustness of the checklist including quality of reporting, internal validity (bias and confounding), external validity, and statistical power [49]. As the authors recognised that many studies included in this review were of observational study design, a modified Downs and Black protocol was used to assess observational studies, similar to methods previously reported [7, 50–53]. The following items were removed that are directed towards intervention studies: items 4, 8, 14, 15, 19, 23, and 24. Items 9, 13, and 22 were modified to also encompass observational studies (Table 2). A dichotomous scoring criterion was utilised for all items (0 = no/unable to determine; 1 = yes), except for item 5, which used a larger scale consistent with the original Downs and Black checklist (0 = no/unable to determine; 1 = partial; 2 = yes) [49]. Scoring for item 27 was modified to a dichotomous scale from the original Downs and Black checklist, which has previously been used in other systematic reviews [50, 51, 54]. These modifications described resulted in a maximum critical appraisal score of 21 points for the assessment of observational studies. The rating scale proposed by Kennelly [55] was then modified to grade the overall methodological quality of each observational study as either poor (≤ 10), fair (11–14), or good (≥ 15), similar to previously published reviews [50, 51]. For intervention studies, a total critical appraisal score out of 28 points was applied, as all initial 27 items remained, with the original Kennelly rating of poor (≤ 14), fair (15–19), and good (≥ 20) utilised [55].

**Table 2:** Modified Downs and Black critical appraisal checklist applied to observational studies (adapted from Downs and Black [49])

Item #	Question
1	Is the hypothesis/aim/objective of the study clearly described?
2	Are the main outcomes to be measured clearly described in the introduction or methods section?
3	Are the characteristics of the participants included in the study clearly described?
4	Removed
5	Are the distributions of principal confounders clearly described?
6	Are the main findings of the study clearly described?
7	Does the study provide estimates of the random variability in the data for the main outcome?
8	Removed
9^*^	Have the characteristics of patients lost to follow up been described or did the study have any participant losses?
10	Have actual probability values been reported for the main outcomes, except where the probability value is < 0.001?
11	Were the subjects asked to participate in the study representative of the entire population from which they were recruited?
12	Were those subjects who were prepared to participate representative of the entire population from which they were recruited?
13^*^	Were the staff, places, and facilities where the participants were treated or where the testing was performed representative of the exams/treatment the majority would receive?
14	Removed
15	Removed
16	If any of the results of the study were based on “data dredging”, was this made clear?
17	In trials and cohort studies, do the analyses adjust for different lengths of follow-up of participants, or in case-control studies, is the time period between the intervention and the outcome the same for cases and controls?
18	Were the statistical tests used to assess the main outcomes appropriate?
19	Removed
20	Were the main outcome measures used accurate (valid and reliable)?
21	Were the participants in different intervention groups (trials and cohort studies) or were the cases and controls (case-control studies) recruited from the same population?
22^*^	Were study subjects recruited over the same period of time?
23	Removed
24	Removed
25	Was there adequate adjustment for confounding in the analyses from which the main findings were drawn?
26	Were losses of patients to follow up taken into account?
27	Did the study have sufficient power to detect a clinically important effect where the probability value for a difference being due to chance is less than 5%?

*Indicates that item number was modified

To further assess risk of bias (ROB) in the included studies, the components acknowledged for observational studies [56] were identified within the internal validity subset items of the modified Downs and Black protocol [49]. The relevant elements included items 16, 18, 20, 21, 22, and 25, producing a possible total score of 6. Low ROB was determined by a total score ≥ 4/6 (≥ 67%). Two reviewers (chief and secondary investigator) independently performed the critical appraisal and ROB analyses and any discrepancies were resolved by a third reviewer.

### Data Synthesis

The summary of evidence was conducted utilising a data synthesis method initially described by Sallis and colleagues [57]. For the premise of this review, a relationship was defined as a reported result investigating the association between a single physical fitness measure and a single measure of sports injury classification. Relationships from both univariate and multivariate analyses were considered and summarised separately. If numerous physical fitness attributes were measured and examined against one or more measure of sports injury within a single study, either individually or within the presence of confounding variables, then each of these relationships was considered separately under univariate or multivariate analyses, respectively. Instances where data were repeated across multiple studies from the same source were only accounted for once in the data synthesis. Additionally, when the same relationship was investigated by more than one univariate analysis statistical method within the same study, the data were only accounted for once in the data synthesis. Finally, when relationships were explored, but the results were reported without any evidence of significance (e.g. *p* value, confidence intervals, or a direct statement regarding significance), they were accounted for in the data synthesis as not associated with sports injury. When relationships were explored and results were plainly not reported, then these relationships were deemed as ‘not reported’ and therefore, not included in the data synthesis.

To synthesise the extracted relationship data from included studies, objective physical fitness measures were allocated into one of the 11 physical fitness attributes and deemed as a significant or not significant relationship associated with sports injury. Whilst performance in some physical fitness measures may be influenced by multiple physical fitness components, each objective physical fitness measure was allocated only once to the most representative physical fitness category using a consensus process between the authors. In multivariate analyses where a single relationship result represented a combination of individual physical fitness measures collectively (regardless if representing either one or multiple physical fitness components), this was synthesised separately as a combined physical fitness category in text (i.e. not in the result tables). A coding system was then implemented to draw conclusions for each physical fitness component or combined physical fitness category and sports injury classification from the body of evidence for both univariate and multivariate analyses. The number of significantly associated relationships divided by the total number of relationships investigated (n/N), multiplied by 100 produced a summary conclusion percentage. To answer the review question, the summary conclusion was classified based on the criteria found in Table 3. These methods have been utilised in previously published reviews [51, 57, 58]. The summative coding percentage was calculated from studies only with a Kennelly [55] rating of ‘fair’ or ‘good’ methodological quality and low ROB to develop conclusions for this systematic review.

**Table 3:** Summary conclusion criteria to synthesise relationship results between physical fitness components and sports injury outcomes (adapted from [57])

Summary conclusion	Criterion
Clear association (consistent result)	≥ 60% of total relationships were deemed significant indicating sufficient evidence to support the significant association between a physical fitness component and sports injury.
Inconsistent association (inconsistent result)	34–59% of total relationships were deemed significant indicating inconsistent evidence to support the association between a physical fitness component and sports injury.
No association (consistent result)	≤ 33% of total relationships were deemed significant indicating sufficient evidence to support no association between a physical fitness component and sports injury.
Unknown result	Less than five relationships were investigated indicating limited evidence provided to support the association between a physical fitness component and sports injury.

Due to the heterogeneity amongst the included studies in this review regarding the physical fitness methodology and variance in injury definitions used, a meta-analysis was not performed. To address the heterogeneity of included studies and their influence on the summary conclusions, the level of evidence was established for each article using definitions adapted to those previously described: (i) level I—RCTs and high-quality prospective cohort studies, (ii) level II—lower quality prospective cohort studies and retrospective cohort studies, (iii) level III—case-control and cross-sectional studies, and (iv) level IV—case series [59, 60]. Level V evidence (expert opinions) did not meet the inclusion criteria for this review. High-quality prospective cohort studies were defined as studies with a Kennelly [55] rating of ‘good’ demonstrating adequate power. If a study did not conduct a power analysis, then 20, 50, or 200 injury cases were required to support strong, moderate, or weak associations, respectively [17]. To encompass the relative strengths of the evidence of individual studies, a level of certainty for each summary conclusion was determined. Definitions of insufficient, low, moderate, and high levels of certainty are outlined in Table 4 and have been adapted from definitions previously modified in other injury risk reviews [59, 60] (see Online Resource 2 for decision-making process for level of certainty).

**Table 4:** Level of certainty definitions used for assessment of summary conclusions (adapted from [59, 60])

Level of certainty	Definition
High	The relationships investigated included evidence from at least two, level I studies with a summary conclusion revealing consistent results. The summary conclusion is unlikely to be strongly affected by future studies.
Moderate	The relationships investigated included evidence from either of the following: (i) only one, level I study and level II and/or level III/IV studies with a consistent summary conclusion; (ii) at least two, level II studies with a consistent summary conclusion; or (iii) level I and/or level II studies with an inconsistent summary conclusion. As more information becomes available, the summary conclusion could change.
Low	The relationships investigated included evidence from either: (i) only one, level II study and level III/IV studies with consistent or inconsistent results; or (ii) level III/IV studies only with consistent or inconsistent results. More information is needed to be certain of the summary conclusion.
Insufficient	The relationships investigated included evidence from only one study (regardless of level of evidence) or with an unknown summary conclusion, indicating < 5 relationships were investigated. More research is needed to establish a relationship summary conclusion.

Consistent result includes a summary conclusion of ‘clear association’ or ‘no association’. Inconsistent result includes a summary conclusion of ‘inconsistent association’

Finally, to explore the contribution of possible confounding variables on the relationships being investigated in this review, the following sub-analyses were conducted to examine the impact on summary conclusions and levels of certainty: (i) competition level (elite versus non-elite) and (ii) chronological age (senior ≥ 18 years old versus junior < 18 years old). The same data synthesis process as described above was implemented for each sub-analysis. Where information regarding the study population relevant to these sub-analyses was not reported, or if the study included combined confounders, these relationships were excluded from the sub-analysis as the data were deemed unable to determine.

## Results

### Study Selection

**Fig. 1:**
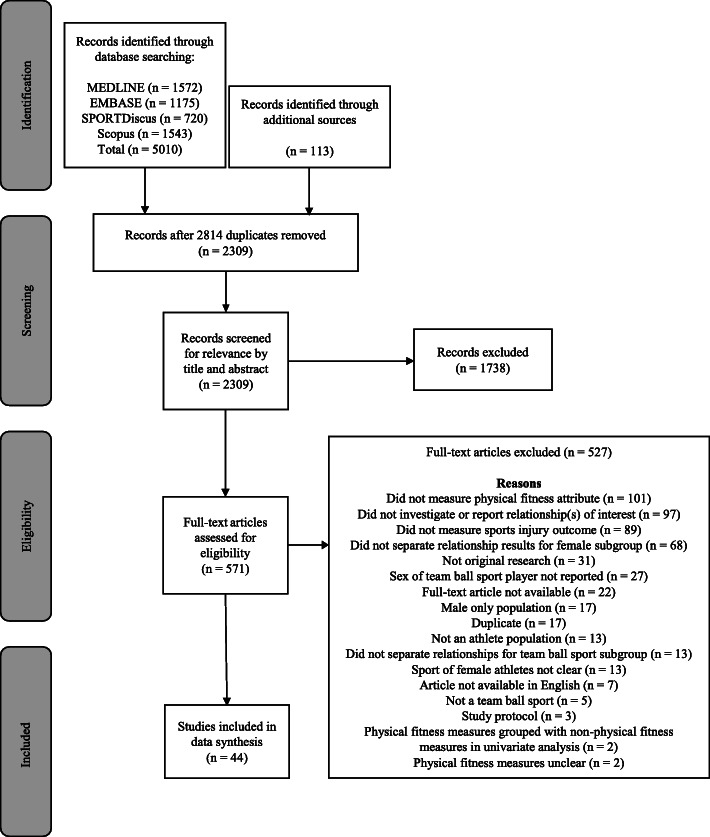
PRISMA flow diagram outlining the search, screening, and selection process

The search produced a total of 5123 records from four databases and handsearching methods, with 2309 studies available for review after duplicates were removed. After screening records for relevance against the eligibility criteria, 44 studies were included in the review (Fig. 1).

### Study Characteristics

Online resource 3 outlines the key data extracted for this review. Of the 44 studies included, the most frequently studied team ball sport was soccer (represented in 24 studies; 55%) [61–84], followed by basketball (represented in 14 studies, 32%) [64, 65, 71, 73, 83, 85–93], and volleyball (represented in 7 studies, 16%) [64, 71, 83, 89–91, 94]. Other team ball sports investigated included handball [81, 90, 95, 96], netball [97–100], softball [101–103], field hockey [61, 65], lacrosse [61], and rugby union [104]. Almost one quarter (23%) of the studies included multiple team ball sport players in their study population.

Sample sizes of the included study populations ranged from 11 to 4556 female participants. The age range of female, team ball sport players was 11 to 26 years of age, with 18 studies (41%) including junior athletes. A variety of competition levels were included from non-elite (27 studies, 61%) to elite (13 studies, 30%), with three studies involving combined groups of non-elite and elite level athletes. One study did not report the competition level of their participants [90]. Study populations were primarily from the US (19 studies; 43%) and Europe (12 studies, 27%). Four studies (9%) did not report the country of origin, with the remaining studies representing five countries spanning four continents (Africa, Asia, Australia, and North America). All studies included in the review were of observational study designs, with the majority being prospective cohort studies (33 studies, 75%). The remaining study designs included cross-sectional studies (8 studies, 18%), case-control studies (2 studies, 5%), and a retrospective cohort study (1 study, 2%).

**Fig. 2:**
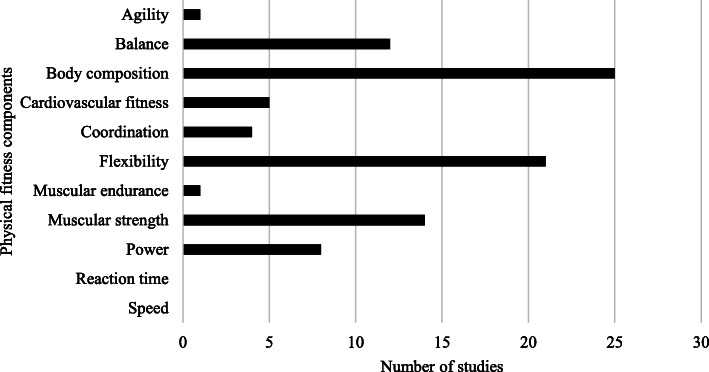
Distribution of physical fitness components captured by studies included in the review

A variety of objective measures were utilised to represent physical fitness components in the included studies. Figure 2 shows the number of studies that examined at least one objective measure representative of a physical fitness attribute and its relationship with sports injury, regardless of methodological quality rating. The physical fitness characteristics of reaction time and speed were not represented in any of the included studies.

A range of sports injury outcomes encompassing various injury definitions and injury types were reported by the reviewed studies. The most common injury definition utilised was time loss (23 studies, 52%), followed by medical attention (12 studies, 27%), and all-complaints (2 studies, 5%) identifiers. Six studies (14%) used a combined injury definition encompassing both time-loss and medical attention identifiers and one study (2%) did not report an injury definition or description for classification. Several studies (33 studies, 75%) used categories (one or several) summarising multiple sports injury types, such as lower extremity injuries, traumatic injuries, or all injuries, whereas 9 studies (20%) examined a single, specific sports injury type, such as ACL injury or ankle sprain. Two studies included a combination of single and multiple sports injury type outcomes. Table 5 demonstrates the number of studies that investigated the relationships between physical fitness risk factors and single and/or multiple sports injury outcome types by univariate and/or multivariate analyses.

**Table 5:** Number and percentage of studies classified by each type of relationship investigation

		Sports injury type	
		Single	Multiple
Risk factors	Single (univariate analysis)	7 studies (16%)	29 studies (66%)
	Multiple (multivariate analysis)	8 studies (18%)	18 studies (41%)

Based on the literature search, the relationship results were divided and synthesised according to the following broad sports injury classifications: (i) ‘any injury’—all-inclusive sports injury classification where the region of the body was not defined; (ii) upper extremity injuries—injuries sustained to the shoulder, elbow, wrist, and/or hand, or collectively classified as upper limb or upper extremity; (iii) lower body injuries—sports injury category reference to the lower quarter or lower extremity with joint region not discriminated, but could be inclusive of the low back and/or pelvis; (iv) thigh/knee injuries—injuries sustained to the thigh and/or knee regions; and (v) lower leg/ankle/foot injuries—injuries sustained to the lower leg, ankle, and/or foot regions. Additionally, those studies that examined a specific, single sports injury type were grouped together and synthesised as a sub-analysis.

### Methodological Quality of Included Studies

The critical appraisal score from the modified Downs and Black checklist [49], modified Kennelly [55] rating, and ROB assessment for each of the included studies is listed in Table 6. A moderate level of agreement between the two reviewers was concluded by Cohen’s kappa analysis (*κ* = 0.490, *p* < 0.001). After a process of consensus with a third reviewer, 100% agreement was achieved for all critical appraisal scores. The number of studies having a ‘good’, ‘fair’, and ‘poor’ methodological quality rating were 12 (27%), 29 (66%), and 3 (7%), respectively. Ten (23%) studies were scored as having high ROB. Areas on the modified Downs and Black checklist [49] that were least represented included all questions for external validity, whether the subjects were recruited over the same period of time, adequate adjustment for confounding in the analyses, and power analysis reported.

**Table 6:** Critical appraisal scores, Kennelly [55] ratings, and ROB assessment based on modified Downs and Black [49]

Study author (year)	Critical appraisal score (out of 21)	Kennelly rating	Risk of bias
Achenbach et al. (2019) [95]	16	Good	Low
Aragon et al. (2012) [101]	15	Good	Low
Armstrong & Greig (2018) [104]	12	Fair	Low
Attenborough et al. (2017) [97]	14	Fair	High
Barber Foss et al. (2012) [85]	13	Fair	High
Beynnon et al. (2001) [61]	14	Fair	Low
Blokland et al. (2017) [62]	14	Fair	Low
Brumitt et al. (2019) [94]	16	Good	Low
Cheng et al. (2019) [63]	12	Fair	Low
Chorba et al. (2010) [64]	14	Fair	Low
Devan et al. (2004) [65]	10	Poor	Low
Edouard et al. (2013) [96]	14	Fair	Low
Emery et al. (2005) [67]	12	Fair	High
Emery & Meeuwisse (2006) [66]	12	Fair	High
Faude et al. (2006) [68]	15	Good	Low
Hägglund & Waldén (2016) [69]	14	Fair	Low
Hill et al. (2004) [102]	9	Poor	High
Hopper et al. (1995) [98]	15	Good	Low
Hopper (1997) [99]	14	Fair	Low
Koenig & Puckree (2015) [70]	14	Fair	Low
Kofotolis & Kellis (2007) [86]	17	Good	Low
Landis et al. (2018) [71]	12	Fair	High
McCann et al. (2018) [72]	14	Fair	High
Myer et al. (2008) [73]	12	Fair	Low
Ness et al. (2017) [74]	13	Fair	Low
Nilstad et al. (2014) [75]	17	Good	Low
Niyonsenga & Phillips (2013) [76]	16	Good	Low
O’Kane et al. (2017) [77]	15	Good	Low
Östenberg & Roos (2000) [78]	14	Fair	Low
Payne et al. [93]	12	Fair	High
Plisky et al. (2006) [87]	16	Good	Low
Räisänen et al. (2018) [79]	16	Good	Low
Shanley et al. (2011) [103]	16	Good	Low
Shimozaki et al. (2018) [88]	13	Fair	High
Smith et al. (2005) [100]	13	Fair	Low
Söderman et al. (2001) [80]	14	Fair	Low
Steffen et al. (2016) [81]	12	Fair	Low
Sugimoto et al. (2018) [82]	12	Fair	Low
van der Worp et al. (2012) [89]	14	Fair	Low
Vauhnik et al. (2008) [90]	13	Fair	Low
Walbright et al. (2017) [91]	12	Fair	High
Warren et al. (2019) [83]	13	Fair	Low
Watson et al. (2017) [84]	14	Fair	Low
Yentes et al. (2014) [92]	10	Poor	Low

Modified Kennelly [55] rating determined by raw critical appraisal score (out of 21) to determine the overall methodological quality of each study as either poor (≤ 10), fair (11–14), or good (≥ 15). Risk of bias rating was determined by internal validity subset items on the Downs and Black checklist [49] (out of 6) as either low (≥ 4) or high (≤ 3)

### Relationships Between Physical Fitness Attributes and Sports injury

#### ‘Any injury’ Classification

Nine studies examined the associations between physical fitness attributes and ‘any injury’ classification by univariate analysis. Injury definitions included time loss and/or medical attention and were inclusive of ‘any injury’ classification obtained during sport. Only one study did not report an injury definition to classify ‘any injury’ [70]. One prospective study (level II) investigated ‘any injury’ regarding total days injured [104], whereas the remaining studies examined any physical injury that occurred during sport using either prospective (level II) [64, 66–68], case-control (level III) [84], or cross-sectional study designs (level III) [70, 76, 102]. Two cross-sectional studies (level III) examined participants’ one-season [102] or three-season [76] history of ‘any injury’. Sports represented included rugby union, soccer, volleyball, basketball, and softball. Three studies were considered to have poor methodological quality and/or high ROB [66, 67, 102].

Five studies conducted multivariate analyses to investigate the relationship between physical fitness and ‘any injury’ classification in female, team ball sport players. Four studies utilised a time-loss injury definition [62, 78, 100, 104] and one study used a medical attention injury identifier [82]. One prospective study (level II) investigated total days injured from ‘any injury’ [104], whereas the remaining prospective studies (levels I and II) examined ‘any injury’ that occurred over one season [62, 78]. Two cross-sectional studies (level III) explored history of ‘any injury’ that occurred during sport [82, 100]. Blokland and colleagues [62] also explored risk factors for any non-contact, recurrent, and match injury classifications. Sports represented by these studies included soccer, netball, and rugby union. All studies were considered to have fair to good methodological quality and low ROB. Table 7 illustrates the summary conclusions and level of certainty of the relationships between physical fitness attributes and ‘any injury’ from univariate and multivariate analyses in studies with a fair to good methodological quality rating and low ROB.

**Table 7:** Summary conclusions and level of certainty from studies examining associations between physical fitness components and ‘any injury’ in female, team ball sport players

Physical fitness components	Relationships assessed from each study		Summary conclusion		Level of certainty
	# relationships demonstrating significant association with injury	# relationships demonstrating no significant association with injury	*n*/*N* relationship outcome (%)	Practical interpretation	High, moderate, low, insufficient
Univariate analyses					
Balance measures	2 [104]	2 [104], 4 [70]	2/8 (25%)	No association	Low
Body composition measures	1 [68]	1 [70], 1 [76], 1 [84], 5 [68]	1/9 (11%)	No association	Low
Cardiovascular fitness measures	1 [84]	1 [84]	1/2 (50%)	Unknown	Insufficient
Coordination measures	1 [64], 2 [104]	1 [64], 12 [104]	3/16 (19%)	No association	Moderate
Flexibility measures	2 [76]	8 [104]	2/10 (20%)	No association	Low
Multivariate analyses					
Agility measures	1 [78]	0	1/1 (100%)	Unknown	Insufficient
Balance measures	4 [104]	0	4/4 (100%)	Unknown	Insufficient
Body composition measures	2 [82]	0	2/2 (100%)	Unknown	Insufficient
Coordination measures	6 [104]	6 [104]	6/12 (50%)	Inconsistent association	Insufficient
Flexibility measures	1 [78], 2 [100], 2 [104]	6 [104], 17 [62]	5/28 (18%)	No association	Moderate

Coding: *n*/*N* = number of significant associated relationships divided by total number of relationships investigated. The number of relationships is reported with the study reference number in brackets

Data removed from univariate analyses data synthesis due to poor methodological quality and/or high ROB included: body composition measures (2 relationships [102]); cardiovascular fitness measures (1 relationship [67], 1 relationship [66]); and power measures (1 relationship [67], 1 relationship [66])

One study investigated two relationships between combined physical fitness components and ‘any injury’ [104]. The individual components of the FMS^TM^ were included together in a multivariate analysis to examine the risk of total days injured from ‘any injury’, and again with contusions removed in female rugby union players [104]. The individual components of the FMS^TM^ represented balance, flexibility, and coordination physical fitness categories. The combined physical fitness components significantly predicted total days injured for all injuries and remained a significant predictor for all injuries when contusions were removed (Online Resource 3). Despite these two significant relationships, the summary conclusion between combined physical fitness measures and sports injury was deemed as ‘unknown’ (2/2, 100%) due to insufficient evidence with an insufficient level of certainty.

Sub-analyses utilising studies with fair to good methodological quality and low ROB revealed no differences in the results when relationships were examined based on chronological age (< 18 years old versus ≥ 18 years old) from univariate or multivariate analyses. Similarly, level of competition (elite versus non-elite) did not influence the results from univariate analyses. However, a change in the summary conclusion from multivariate analyses occurred from ‘no association’ to an ‘inconsistent association’ (4/10, 40%) when examined the relationship between flexibility and ‘any injury’ category for non-elite ball players. Nevertheless, this result was deemed with a low level of certainty. No studies with fair to good methodological quality and low ROB reported independent associations by univariate or multivariate analysis between, muscular endurance, muscular strength, power, reaction time, or speed attributes and ‘any injury’ classification. Additionally, no studies with fair to good methodological quality and low ROB reported associations between agility or cardiovascular fitness, by univariate and multivariate analysis, respectively, and ‘any injury’ classification.

#### Upper Extremity Injuries

Four studies investigated the relationship between physical fitness attributes and upper extremity injuries by univariate analysis. Two studies (level II) utilised an all-complaints definition to prospectively examine any upper extremity injury [103] or overuse shoulder injuries [95]. The remaining two studies used a time-loss injury definition to investigate prospective (level II) shoulder injuries [96] or history of shoulder or elbow injuries by cross-sectional study design (level III) [101]. Both handball and softball players comprised study populations. All studies were considered to have fair to good methodological quality and low ROB. Data synthesis of associations between physical fitness attributes and upper extremity injuries by univariate analyses from studies with fair to good methodological quality and low ROB is reported in Table 8. No studies performed a multivariate analysis including physical fitness variables to understand their relationship with upper extremity injury in female, team ball sport players.

**Table 8:** Summary conclusions and level of certainty from studies examining associations between physical fitness components and upper extremity injury in female, team ball sport players

Physical fitness components	Relationships assessed from each study		Summary conclusion		Level of certainty
	# relationships demonstrating significant association with injury	# relationships demonstrating no significant association with injury	*n*/*N* relationship outcome (%)	Practical interpretation	High, moderate, low, insufficient
Univariate analyses					
Flexibility measures	2 [95], 2 [101]	4 [101], 10 [103]	4/18 (22%)	No association	Moderate
Muscular strength measures	3 [96]	1 [95], 7 [96]	3/11 (27%)	No association	Moderate

Coding: *n*/*N* = number of significant associated relationships divided by total number of relationships investigated. The number of relationships is reported with the study reference number in brackets

Sub-analyses revealed no differences in the results when relationships were examined based on level of competition (elite versus non-elite) or chronological age (< 18 years old versus ≥ 18 years old) from univariate analyses. No studies with fair to good methodological quality and low ROB reported independent associations by univariate analysis between agility, balance, body composition, cardiovascular fitness, muscular endurance, muscular strength, power, reaction time, or speed attributes and upper extremity injuries.

#### Lower Body Injuries

Thirteen studies investigated the relationship between physical fitness attributes and lower body injuries by univariate analysis in basketball, soccer, volleyball, and netball players. Time-loss injury definition was used by eight studies [74, 75, 77, 80, 87, 91, 92, 94], three studies defined injury by medical attention [83, 98, 99], and two studies utilised both time-loss and medical attention injury definitions [71, 79]. Most studies investigated lower body injuries prospectively (levels I and II), and were categorised as any injury to the lower quarter [91], lower extremity [75, 87, 92], or low back/lower extremity [98]; acute [79] or traumatic [80] injury to the lower extremity; noncontact injuries to the lower extremity injury [71, 79] or low back/lower extremity [83, 94]; or overuse lower extremity injuries [77, 80]. The remaining two studies were retrospective cohort (level II) [74] and cross-sectional (level III) [99] in design and used any lower extremity or low back/lower extremity injury classifications, respectively. Three studies were considered to have poor methodological quality and/or high ROB [71, 91, 92].

The relationship between physical fitness attributes and lower body injuries was investigated by multivariate analyses from eight prospective cohort (levels I and II) and one retrospective cohort (level II) studies. Five studies utilised a time-loss injury definition [63, 74, 75, 80, 87], two studies used a medical attention injury definition [83, 98], and the remaining two studies used a combination of both medical attention and time-loss injury identifiers [71, 79]. Lower body injuries were classified as any injury to the lower body [63], lower extremity [74, 75, 87] or low back/lower extremity [98]; acute [79] or traumatic [80] injuries to the lower extremity; noncontact injuries to the lower extremity [71, 79] or low back/lower extremity [83]; or overuse injuries to the lower extremity [80] or lower body [63]. Cheng and colleagues [63] also investigated lumbopelvic, hip, incomplete recovery of lower body injury, and multiple lower body injuries categories separately. Study populations consisted primarily of soccer players, with basketball, volleyball, and netball players also represented. One study was considered to have high ROB [71]. Table 9 outlines the summary conclusions and level of certainty of the relationships between physical fitness characteristics and lower body injuries from univariate and multivariate analyses with fair to good methodological quality and low ROB.

**Table 9:** Summary conclusions and level of certainty from studies examining associations between physical fitness components and lower body injury in female, team ball sport players

Physical fitness components	Relationships assessed from each study		Summary conclusion		Level of certainty
	# relationships demonstrating significant association with injury	# relationships demonstrating no significant association with injury	*n*/*N* relationship outcome (%)	Practical interpretation	High, moderate, low, insufficient
Univariate analyses					
Balance measures	1 [80], 9 [87]	4 [98], 3 [87], 3 [80], 1 [75]	10/21 (48%)	Inconsistent association	Moderate
Body composition measures	1 [98], 2 [75]	1 [75], 2 [74], 3 [99], 4 [98], 6 [79]	3/19 (16%)	No association	Moderate
Cardiovascular fitness measures	1 [74]	0	1/1 (100%)	Unknown	Insufficient
Flexibility measures	3 [80]	1 [98], 1 [75], 1 [77], 5 [80]	3/11 (27%)	No association	Moderate
Muscular strength measures	1 [83], 2 [80]	2 [79], 2 [80], 4 [75], 5 [83], 7 [77]	3/23 (13%)	No association	Moderate
Power measures	1 [83], 4 [98]	2 [83], 4 [94]	5/11 (45%)	Inconsistent association	Moderate
Multivariate analyses					
Balance measures	1 [80], 1 [87]	8 [87]	2/10 (20%)	No association	Moderate
Body composition measures	1 [75], 1 [98]	2 [79], 2 [74]	2/6 (33%)	No association	Moderate
Cardiovascular fitness measures	1 [74]	0	1/1 (100%)	Unknown	Insufficient
Flexibility measures	0	6 [63]	0/6 (0%)	No association	Insufficient
Muscular strength measures	1 [80], 1 [83]	1 [79], 5 [83]	2/8 (25%)	No association	Moderate
Power measures	1 [83], 1 [98]	2 [83], 3 [98]	2/7 (29%)	No association	Moderate

Coding: *n*/*N* = number of significant associated relationships divided by total number of relationships investigated. The number of relationships is reported with the study reference number in brackets

Data removed from univariate analyses’ data synthesis due to poor methodological quality and/or high risk of bias included the following: muscular strength measures (6 relationships [92]), flexibility measures (4 relationships [91]), balance measures (10 relationships [91]), coordination measures (1 relationship [71], 9 relationships [91]), and power measures (2 relationships [91]). Data removed from multivariate analyses’ data synthesis due to poor methodological quality and/or high risk of bias included the following: body composition measures (2 relationships [71]), balance measures (1 relationship [71]), coordination measures (6 relationships [71]), and flexibility measures (2 relationships [71])

One prospective cohort (level I) study examined whether combined power measures, as assessed by standing long jump, bilateral single leg hop for distance, and single leg hop side-to-side asymmetry, were associated with noncontact low back or lower extremity injury in female, collegiate volleyball players [94]. Results concluded those with suboptimal standing long jump and bilateral single leg hop for distance scores and > 10% single leg hop asymmetry were four times more likely to have a noncontact back or lower extremity injury; however, when the single leg hop asymmetry parameter was removed, this second relationship was not significant [94] (Online Resource 3). As only two relationships investigated the association between combined power measures and lower body injuries, the summary conclusion was considered as ‘unknown’ (1/2, 50%) with insufficient level of certainty.

Sub-analyses of univariate analyses revealed a ‘clear association’ between balance and lower body injury in non-elite (10/16, 63%) and junior (9/12, 75%) team ball sport players, with moderate and insufficient levels of certainty, respectively. ‘No association’ (1/9, 11%) was concluded for senior participants from univariate analyses. Most significant relationships revealed those players with poor balance were at an increased risk of lower body injury. Sub-analysis also produced ‘no association’ (1/7, 14%) between power measures and lower body injury with moderate level of certainty and an ‘inconsistent association’ (3/8, 38%) between flexibility attributes and lower body injury with insufficient certainty in non-elite team ball sport players. Sub-analyses of multivariate studies utilising studies with fair to good methodological quality and low ROB revealed no differences in the results when relationships were examined based on level of competition (elite versus non-elite) or chronological age (< 18 years old versus ≥ 18 years old). No studies with fair to good methodological quality and low ROB reported associations by univariate or multivariate analyses between agility, coordination, muscular endurance, reaction time, or speed physical fitness components and lower body injury.

#### Thigh/Knee Injuries

The relationship between physical fitness measures and thigh/knee injuries from univariate analyses was investigated in nine studies. All but one case-control (level III) study [73] implemented a prospective cohort study design (levels I and II). Time-loss injury definition was used in six studies [69, 75, 77, 85, 90, 94], two studies utilised a medical attention definition [65, 73], and one study incorporated both to define injuries [71]. Injuries were classified as any injury to the thigh or knee [75]; acute [69] or traumatic [90] knee injuries; injury to the ACL [69, 73], including noncontact mechanism only [71]; noncontact injuries to the thigh/knee [94]; overuse knee injuries [65, 77]; or patellofemoral pain [85]. Sports represented in study populations included basketball, handball, volleyball, soccer, and field hockey players. Three studies were classified as having poor methodological quality or high ROB [65, 71, 85].

Twelve studies examined the relationship between physical fitness attributes and thigh/knee injuries by multivariate analyses. All but two studies were prospective (levels I and II) in study design, with the remaining a case-control study (level III) [73] and cross-sectional (level III) in nature [89]. Seven studies utilised a time-loss injury definition [62, 63, 69, 75, 78, 85, 90], four studies used a medical attention injury definition [73, 81, 88, 89], and the remaining study used a combination of both time-loss and medical attention injury identifiers [71]. Thigh/knee injuries were classified as any injury to the thigh [62, 75] or knee [62, 63, 75, 78]; acute [69] or traumatic [90] knee injuries; injury to the ACL [69, 73], including noncontact mechanism only [71, 81, 88]; or diagnoses of patellofemoral pain [85] or patellar tendinopathy [89]. Soccer, basketball, and handball players were represented in the study populations. High ROB was concluded for three studies [71, 85, 88]. Table 10 shows the summary conclusions and level of certainty of the relationships from studies with fair to good methodological quality and low ROB that investigated the association between physical fitness measures and thigh/knee injuries by univariate and multivariate analyses.

**Table 10:** Summary conclusions and level of certainty from studies examining associations between physical fitness components and thigh/knee injuries in female, team ball sport players

Physical fitness components	Relationships assessed from each study		Summary conclusion		Level of certainty
	# relationships demonstrating significant association with injury	# relationships demonstrating no significant association with injury	*n*/*N* relationship outcome (%)	Practical interpretation	High, moderate, low, insufficient
Univariate analyses					
Balance measures	0	2 [75]	0/2 (0%)	Unknown	Insufficient
Body composition measures	1 [69]	1 [69], 2 [75], 3 [90]	1/7 (14%)	No association	Moderate
Flexibility measures	0	1 [77], 1 [90], 2 [75], 4 [73]	0/8 (0%)	No association	Moderate
Muscular strength measures	4 [77]	3 [77], 8 [75]	4/15 (27%)	No association	Moderate
Power measures	0	4 [94]	0/4 (0%)	Unknown	Insufficient
Multivariate analyses					
Body composition measures	1 [75], 1 [90]	2 [69], 2 [89]	2/6 (33%)	No association	Moderate
Flexibility measures	1 [73], 1 [78]	1 [63], 2 [73], 7 [62]	2/12 (17%)	No association	Moderate
Muscular strength measures	0	1 [75], 5 [81]	0/6 (0%)	No association	Moderate

Coding: *n*/*N* = number of significant associated relationships divided by total number of relationships investigated. The number of relationships is reported with the study reference number in brackets

Data removed from univariate analyses’ data synthesis due to poor methodological quality and/or high risk of bias included the following: body composition measures (3 relationships [85]), muscular strength (2 relationships [65]), muscular endurance measures (2 relationships, [65]), and coordination measures (1 relationship [71]). Data removed from multivariate analyses’ data synthesis due to poor methodological quality and/or high risk of bias included the following: balance measures (1 relationship [71], 1 relationship [88]), body composition measures (1 relationship [88], 2 relationships [71]), coordination measures (5 relationships [71]), flexibility (1 relationship [88], 2 relationships [71]), and muscular strength measures (3 relationships [88])

Similar to lower body injuries, the same prospective study (level I) examined whether combined power measures (standing long jump, bilateral single leg hop for distance, and single leg hop side-to-side asymmetry) were associated with prospective, noncontact thigh/knee injuries in female, collegiate volleyball players [94]. No significant results were found [94] (Online Resource 3) and the summary conclusion revealed as ‘unknown’ (0/2, 0%), as < 5 relationships were reported, with insufficient level of certainty.

Sub-analysis revealed an ‘inconsistent association’ (4/7, 57%) between muscular strength and thigh/knee injury from univariate analyses in female, junior team ball sport players. Four significant relationships were reported that demonstrated those players with decreased hip flexor, hip external rotation, quadriceps, and hamstring strength had an increased risk of overuse knee injuries; however, hip extensor, abductor, and adductor strength were not significantly associated [77] (Online Resource 3). However, this summary conclusion had an insufficient level of certainty as results were synthesised from only one study. No differences in univariate results were found when analysed by competition level. Sub-analyses revealed no differences in the results when relationships were examined based on level of competition (elite versus non-elite) or chronological age (< 18 years old versus ≥ 18 years old) from multivariate analyses. No studies with fair to good methodological quality and low ROB reported independent associations by univariate or multivariate analyses between agility, cardiovascular fitness, coordination, muscular endurance, reaction time, or speed physical fitness components and thigh/knee injuries. Additionally, no studies with fair to good methodological quality and low ROB reported relationships by multivariate analysis between balance and thigh/knee injury.

#### Lower Leg/Ankle/Foot Injuries

Five studies examined the relationship between physical fitness characteristics and lower leg/ankle/foot injuries by univariate analyses. All studies were prospective (levels I and II) in design and defined injury as either time-loss [75, 94, 97], medical attention [61], or combined time-loss and medical attention definitions [72]. Lower leg/ankle/foot injuries encompassed any injury to the ankle or leg/foot [75], noncontact injuries to the ankle/foot [94], or diagnosis of an ankle sprain [61, 72, 97]. Sports represented in study populations included volleyball, soccer, field hockey, lacrosse, and netball. Two studies were identified as having high ROB [72, 97].

The relationship between physical fitness attributes and lower leg/ankle/foot injuries by multivariate analyses was examined in by four studies. All studies were prospective (level II) in study design and utilised a time-loss or combined time-loss and medical attention injury definitions. Lower leg/ankle/foot injuries were categorised as any injury to the ankle [62, 75, 93] or leg/foot [75], or an ankle sprain diagnosis [86]. Basketball and soccer players were represented by two studies each. All but one study [93] were considered to have fair to good methodological quality with low ROB. Table 11 illustrates the summary conclusion and level of certainty of the relationships between physical fitness attributes and lower leg/ankle/foot injuries from univariate and multivariate analyses with fair to good methodological quality and low ROB.

**Table 11:** Summary conclusions and level of certainty from studies examining associations between physical fitness components and lower leg/ankle/foot injuries in female, team ball sport players

Physical fitness components	Relationships assessed from each study		Summary conclusion		Level of certainty
	# relationships demonstrating significant association with injury	# relationships demonstrating no significant association with injury	*n*/*N* relationship outcome (%)	Practical interpretation	High, moderate, low, insufficient
Univariate analyses					
Balance measures	0	2 [75], 3 [61]	0/5 (0%)	No association	Moderate
Body composition measures	0	2 [61], 2 [75]	0/4 (0%)	Unknown	Insufficient
Flexibility measures	1 [61]	2 [75], 4 [61]	1/7 (14%)	No association	Moderate
Muscular strength measures	1 [75]	7 [75], 12 [61]	1/20 (5%)	No association	Moderate
Power measures	0	4 [94]	0/4 (0%)	Unknown	Insufficient
Multivariate analyses					
Body composition measures	0	1 [75], 2 [86]	0/3 (0%)	Unknown	Insufficient
Flexibility measures	0	3 [62]	0/3 (0%)	Unknown	Insufficient
Muscular strength measures	0	1 [75]	0/1 (0%)	Unknown	Insufficient

Coding: *n*/*N* = number of significant associated relationships divided by total number of relationships investigated. The number of relationships is reported with the study reference number in brackets

Data removed from univariate analyses data synthesis due to poor methodological quality and/or high risk of bias included the following: body composition measures (3 relationships [72], 2 relationships [97]), flexibility measures (1 relationship [97]), balance measures (5 relationships [97]), and power measures (1 relationship [97]). Data removed from multivariate analyses’ data synthesis due to poor methodological quality and/or high risk of bias included: flexibility measures (2 relationships [93]) and muscular strength measures (16 relationships [93])

Brumitt and colleagues [94] (level I prospective cohort study) also examined whether combined power measures (standing long jump, bilateral single leg hop for distance, and single leg hop side-to-side asymmetry) were associated with noncontact ankle/foot injury in female, collegiate volleyball players. Similar results were found as in the lower body injury category in that those players with suboptimal power performance were 6 times more likely to experience injury; however, this relationship was no longer significant when the single leg hop side-to-side asymmetry measure was removed [94] (Online Resource 3). Therefore, the summary conclusion for combined power measures and risk of lower leg/ankle/foot injuries is deemed as ‘unknown’ (1/2, 50%) with insufficient level of certainty.

Sub-analyses revealed no differences in the results when relationships were examined based on level of competition (elite versus non-elite) or chronological age (< 18 years old versus ≥ 18 years old) from univariate or multivariate analyses. No studies with fair to good methodological quality and low ROB reported associations by univariate or multivariate analyses between agility, cardiovascular fitness, coordination, muscular endurance, reaction time, or speed attributes and lower leg/ankle/foot injuries. Additionally, no studies with fair to good methodological quality and low ROB investigated relationships by multivariate analysis between balance and power measures and lower leg/ankle/foot injury.

#### Single Injury Types

Sub-analysis revealed eleven studies investigated the relationship between physical fitness measures and single injury types by univariate and/or multivariate analyses. The literature search revealed the following single injury types: ACL injury in soccer and/or basketball players [69, 73], noncontact ACL injuries in handball, soccer, basketball, and/or volleyball players [71, 81, 88], patellar tendinopathy in basketball and volleyball players [89], patellofemoral pain in basketball players [85], ankle sprains in lacrosse, soccer, field hockey, basketball, and/or netball players [61, 86, 97], and lateral ankle sprains in soccer players [72]. Injury definitions were almost evenly split between medical attention [61, 73, 81, 88, 89] and time-loss [69, 85, 86, 97] identifiers, with two studies using a combined medical attention and time-loss injury definition [71, 72]. Nine studies implemented prospective cohort designs (level II) [61, 69, 71, 72, 81, 85, 86, 88, 97], with the remaining two studies of cross-sectional (level III) [89], and case-control (level III) [73] in nature. Five studies were considered to have poor methodological quality and/or high ROB [71, 72, 85, 88, 97]. Table 12 shows the summary conclusions and level of certainty from univariate and multivariate analyses with fair to good methodological quality and low ROB of the relationships between physical fitness attributes and the following single injury types: ACL injury, noncontact ACL injury, patellar tendinopathy, and ankle sprains. Patellofemoral pain and lateral ankle sprains were not included in the results’ table as all relationships were from studies with high ROB.

**Table 12:** Summary conclusions and level of certainty from studies examining associations between physical fitness components and single injury type

Physical fitness components	Relationships assessed from each study		Summary conclusion		Level of certainty
	# relationships demonstrating significant association with injury	# relationships demonstrating no significant association with injury	*n*/*N* relationship outcome (%)	Practical interpretation	High, moderate, low, insufficient
*ACL injury*					
Univariate analyses					
Body composition measures	0	1 [69]	0/1 (0%)	Unknown	Insufficient
Flexibility measures	0	4 [73]	0/4 (0%)	Unknown	Insufficient
Multivariate analyses					
Body composition measures	0	1 [69]	0/1 (0%)	Unknown	Insufficient
Flexibility measures	1 [73]	2 [73]	1/3 (33%)	Unknown	Insufficient
*Noncontact ACL injury*					
Multivariate analyses					
Muscular strength measures	0	5 [81]	0/5 (0%)	No association	Insufficient
*Ankle sprain*					
Univariate analyses					
Balance measures	0	3 [61]	0/3 (0%)	Unknown	Insufficient
Body composition measures	0	2 [61]	0/2 (0%)	Unknown	Insufficient
Flexibility measures	1 [61]	4 [61]	1/5 (20%)	No association	Insufficient
Muscular strength measures	0	12 [61]	0/12 (0%)	No association	Insufficient
Power measures	0	0	0/0 (0%)	Unknown	Insufficient
Multivariate analyses					
Body composition measures	0	2 [86]	0/2 (0%)	Unknown	Insufficient
*Patellar tendinopathy*					
Multivariate analyses					
Body composition measures	0	2 [89]	0/2 (0%)	Unknown	Insufficient

Coding: *n*/*N* = number of significant associated relationships divided by total number of relationships investigated. The number of relationships is reported with the study reference number in brackets

Data removed from univariate analyses’ data synthesis due to poor methodological quality and/or high ROB included: noncontact ACL injury—coordination measures (1 relationship [71]), ankle sprain—balance measures (5 relationships [97]), body composition measures (2 relationships [97]), flexibility measures (1 relationship [97]), and power measures (1 relationship [97]). Data removed from multivariate analyses’ data synthesis due to poor methodological quality and/or high ROB included: noncontact ACL injury—balance (1 relationship [88], 1 relationship [71]), body composition measures (1 relationship [88], 2 relationships [71, 102]), coordination measures (5 relationships [71]), flexibility measures (1 relationship [88], 2 relationships [71]), muscular strength measures (3 relationships [88])

Sub-analyses revealed no differences in the results when relationships were examined based on level of competition (elite versus non-elite) or chronological age (< 18 years old versus ≥ 18 years old) from univariate or multivariate analyses. No studies with fair to good methodological quality and low ROB reported associations by univariate or multivariate analyses between agility, cardiovascular fitness, coordination, muscular endurance, reaction time, or speed attributes and single injury types.

## Discussion

The aim of this systematic review was to investigate if physical fitness attributes were associated with injury in female, team ball sport players. Findings consistently concluded no association between flexibility or muscular strength physical fitness components and sports injury categorised by body regions (lower body, thigh/knee, lower leg/ankle/foot, and upper extremity), as well as between flexibility and ‘any injury’ classification, with predominantly moderate certainty. No associations were identified between body composition measures and ‘any injury’, lower body, and thigh/knee injury categories, with low to moderate levels of certainty. Furthermore, no association was found between balance and ‘any injury’ and lower leg/ankle/foot injury, demonstrating low and moderate certainty of these findings, respectively. Mixed summary conclusions were demonstrated between balance and lower body injury with moderate certainty of these results. Sub-analyses did, however, reveal a clear association with moderate certainty between balance and lower body injuries in non-elite players. This finding was also revealed in junior players, but with insufficient certainty. Finally, no associations were found between strength and noncontact ACL injuries and ankle sprains, as well as between flexibility and ankle sprains; however, insufficient certainty was identified for these summary conclusions. These main findings implicate both scientists and practitioners working with female, team ball sport players, particularly due to the lack of research regarding the direct role of physical fitness and its association with sports injury in this population.

Despite significant relationships demonstrating players with increased joint mobility, joint laxity, or muscular flexibility were at an increased risk of sports injury [61, 73, 76, 78, 80, 95, 100, 101, 104], no association summary conclusions with primarily moderate certainty were consistently demonstrated between flexibility and all injury categories in female, team ball sport players. The lack of association between flexibility and sports injury supports the results of several studies that have included stretching exercises in injury prevention programs, but found no significant reduction in injury rates [105–107]. In contrast, a systematic review reported moderate evidence that decreased hamstring and ankle flexibility were associated with increased risk of a musculoskeletal injury in military and sports populations [108]. However, the summary conclusions in this review [108] were drawn from mostly military personnel and male athletes, with female, team ball sport players only represented in one study that also included individual racquet sports and fencing athletes. Therefore, the differences in results between reviews may possibly be due to some research showing females have greater flexibility than their male counterparts [109, 110].

Significant relationships captured by this review demonstrated inconsistent results of increased strength [75], decreased strength [77, 83], or greater muscular strength imbalances [80, 96] to have an increased risk of sports injury in female, ball sport players. Despite these reported significant relationships, the collective available evidence suggests no association with moderate certainty exists between muscular strength and all body region injury categories. Other systematic reviews investigating the relationship between specific injuries and muscular strength produced conflicting results in predominately male athletes. For example, decreased hip adductor strength was found to be a consistent risk factor for groin injury in sport [13], whereas an increased quadricep torque was associated with an increased risk of hamstring muscle strains [7]. Additionally, a recent review summarises the growing evidence suggesting eccentric hamstring strength protects against hamstring strain injuries in elite athletes [111]. Conversely, inconclusive evidence for muscle imbalance as a risk factor of injury has been determined, as insufficient evidence was available to support the use of isokinetic muscle testing as a screening test to support common practices in male premier soccer leagues [112]. Similarly, there was limited evidence to support the association between isoinertial muscle testing and musculoskeletal injury risk in military and athletic populations, whereas a moderate association was reported between isometric muscle testing and musculoskeletal injury risk; however, the direction of association was not determined [113]. Whilst the summary conclusion from this review was deemed as no association between muscular strength and sports injury, a moderate level of certainty combined with these inconsistent findings in the literature highlights the need for high-quality research in homogeneous populations to better our understanding of muscle strength and its association with sports injury.

In light of significant relationships demonstrating taller ball players [68, 90] or those with a greater BMI [69, 75, 82] had an increased sports injury risk, the collective evidence found body composition (inclusive of anthropometric measures) was not associated with ‘any injury’, lower body, or thigh/knee injury classifications. Similar findings have been noted regarding a lack of association between risk factors, such as weight, BMI, height, and body fat percentage, with groin injury [13], patellofemoral pain [114], and hamstring strains [7]. In contrast to the lack of associations between body composition and injury in this review, an area of emerging research suggests that relationships between the change of anthropometric characteristics, indicating growth and maturation, and injury may be of importance in youth athletes [115–117]. Whilst the findings in this review revealed predominantly no association between body composition and sports injury, the levels of certainty were low to moderate, indicating more information is needed. Additionally, understanding the influence of skeletal maturity could be an important confounding factor in advancing our knowledge of the incidence of sports injuries in adolescent female, team ball sport players.

No associations were also concluded between balance and ‘any injury’, lower body, and lower leg/ankle/foot injury classifications, with low to moderate certainty. However, sub-analyses of univariate investigations concluded a clear association with moderate certainty between balance and lower body injuries in female, non-elite team ball sport players. Nevertheless, the direction of this relationship was somewhat mixed. One study reported nine relationships whereby female, non-elite junior basketball players with decreased or asymmetrical balance ability (as measured by the Y-balance test) were at an increased risk of experiencing a lower extremity injury [87]. Conversely, one relationship demonstrated female soccer players who had higher balance scores (indicating higher postural sway) had a protective effect on injury risk [80]. In other words, those players who had poorer balance were at a decreased risk of traumatic leg injuries. Given the recordable injury event was a traumatic leg injury, a possible explanation for this finding could be that those with better balance were also more talented players and therefore, may play more aggressively and are exposed to situations where a traumatic leg injury may emerge [80]. Such a result is consistent with the finding that female, non-elite junior soccer players with greater skill have been shown to be at greater risk of injury than their less skilled teammates [118]. Additionally, these conflicting significant relationship results could be explained by the methods used to assess balance, with the first study assessing balance ability of a single limb during movement of the body [87] and the latter investigating postural sway during a one-legged stance on unstable surface [80]. Thus, these measures of balance could be categorised as dynamic and static balance measures, respectively [119]. A lack of correlation between static and dynamic balance performance has been demonstrated in healthy, physically active adults, indicating differences in demands required to maintain postural stability in these tasks [120]. Additionally, the dynamic nature of the Y-balance test also requires lower extremity strength, range of motion, and coordination [87]. This highlights how the interaction of multiple physical fitness components is often required to successfully produce a movement [121]. In addition to the impact of the injury definition used, highlighting that the choice of definition matters [47] and the heterogeneity of methods performed to measure physical fitness, this summary conclusion was also drawn from synthesis of univariate analyses, and therefore disregards the impact of confounding factors. Thus, the association between balance and lower body injuries in female, non-elite team ball sport players should be interpreted with caution and more research is required to clarify these results.

Finally, sub-analysis of investigations on the relationships between physical fitness attributes and single injury types revealed no associations between strength and noncontact ACL injury and ankle sprains; however, the levels of certainty were deemed as insufficient and therefore, these summary conclusions should be interpreted with caution. Nonetheless, of interest is the insufficient certainty result, indicating a lack of evidence demonstrating the relationships between muscular strength and noncontact ACL injuries and ankle sprains. Particularly, no significant relationships were reported for either injury outcome. The authors found this result surprising, given systematic reviews have demonstrated decreased ACL injury [105] and ankle sprain [122] risk when interventions included muscular strength exercises as part of neuromuscular training programs. Neuromuscular training typically involves multimodal training, such as balance, strength, power, and agility exercises [123] and is a common topic in injury prevention research in female athletes [105, 124–126]. Yet, despite this plethora of injury prevention research including these physical fitness components in their interventions, the results from this systematic review indicate a gap in the literature exists in demonstrating the relationship between physical fitness and injury.

Whilst the findings from this review revealed little associations between physical fitness components and sports injury in our target population, we should not conclude that physical fitness testing should not be implemented by sport practitioners. Rather, measuring physical fitness characteristics could offer practitioners insight into game or sport demands [127, 128], identify areas for continued athletic development [129, 130], and be useful as a baseline measure to quantify the effectiveness of a training program across different phases of the sport season [129, 130] or return-to-play decision-making [131].

### Strengths and Limitations

A comprehensive search strategy and systematic screening approach was utilised in this review [132]. Additionally, an extensive critical appraisal of methodological quality with ROB assessment of included studies was performed to strengthen synthesised conclusions. However, a number of limitations of the included literature and the review process may influence findings. Firstly, the studies captured here were all observational. It is therefore important to note that this review only addressed potential causal relationships at best, and not predictive, which is just the first step to help understand why injuries occur [18]. Whilst some studies included in this review implemented multivariate statistical techniques to explore potential confounders, few demonstrated statistical power or captured enough injury cases to detect moderate to strong or small to moderate associations [17]. Therefore, studies that did not utilise a multivariate statistical technique or those lacking sufficient statistical power may result in biased significant (or not significant) relationships reported.

Only a portion of team ball sports searched were represented in the included study populations. Additionally, many studies were classified as having poor methodological quality and/or high ROB. Both limitations indicate a lack of high-quality research in physical fitness injury risk factors in female, team ball sport players, which may explain the large number of ‘unknown’ summary conclusions found in this review. Additionally, it also is important to note those summary conclusions with insufficient or low levels of certainty, indicating more research is needed to better understand these relationships.

Common challenges researchers’ face in injury epidemiology research are the variety of injury definitions and methods utilised to record injured cases implemented, thereby potentially impacting outcomes and subsequent understanding of injury in an athlete population [133–135]. A noteworthy limitation therefore includes the summary conclusions synthesised from this systematic review were collated from primarily grouped injury classifications apparent in the included studies, regardless of injury definition or injury surveillance methods utilised. Additionally, the authors’ decision to only include articles published in English language is a limitation to note, as some relevant literature may have been missed.

Finally, only isolated physical fitness factors and their relationship with injury were examined here. Whilst little association was found, other potential risk factors contributing to the multifactorial nature of sports injury were not included. It is important to note that there is considerable research reviewing the anatomical and biomechanical factors and their relationship with sports injury, particularly regarding ACL injury [15, 136–139]. One explanation of the no association findings in this review and the promising effects of reduction in injury risk with intervention studies may be that physical fitness may not have a direct influence on injury risk, but rather an interaction with other influencing components. Therefore, it is recommended that future research investigates the relationships between injury determinants from the viewpoint of a complex system to better understand the emergence of sports injury [140]. This research should focus on investigating the athlete as a complex system by understanding the interactions of various injury determinants (e.g. physical fitness, biomechanical, psychological, training characteristics, etc.) and their influence on a risk profile, which then produces an emerging pattern (i.e. injury), rather than isolated linear relationships between the determinants themselves and injury [140]. Achievement of this may be enhanced by individual studies publishing their datasets for researchers to work collectively, rather than continuing to conduct small, low quality studies that minimally advance our understanding of this multifaceted problem.

## Conclusion

The present systematic review found that most physical fitness components were not associated with sports injury (moderate certainty) in female, team ball sport players. Only one, clear association was demonstrated between balance and lower body injuries in female, non-elite team ball sport players, most likely suggesting those with poorer balance ability may be at increased risk for injury to the lower limb (moderate certainty). The majority of relationships between physical fitness components and sports injury were ‘unknown’ or with ‘insufficient certainty’ due to insufficient evidence, indicating limited, high-quality published studies were available to demonstrate relationships in female, team ball sport players. The lack of associations is possibly due to the reductionist methods of examining sports injury risk. High-quality, holistic evidence investigating the multifactorial nature of sports injury in female, team ball sport players is required. Specifically, research investigating the interactions that physical fitness attributes have with other injury determinants and how this changes over time would be valuable to better understand the role of physical fitness in the complex system of sports injuries in female, team ball sport players.

## Supplementary information

**Additional file 1.** MEDLINE search strategy.

**Additional file 2.** Level of certainty decision-making flow chart.

**Additional file 3.** Data extraction table including characteristics of studies included in the review.

## Data Availability

Not applicable.

## Abbreviations

ACL: Anterior cruciate ligament
MeSH: Medical subject heading
PRISMA: Preferred Reporting Items for Systematic Reviews and Meta-analysis
ROB: Risk of bias
RCT: Randomised control trial
