# Supplementary material for: The relationship between physical fitness attributes and sports injury in female, team ball sport players: a systematic review
Source: Sports Med Open. 2020 Sep 14;6:45. doi: 10.1186/s40798-020-00264-9 (PMC7490320; doi:10.1186/s40798-020-00264-9)
Supplement: Supplementary file 1 — Additional file 1. MEDLINE search strategy. [file 40798_2020_264_MOESM1_ESM.pdf]

Online Resource 1

MEDLINE search strategy used for the systematic review titled, "The relationship between physical fitness attributes and sports injury in female, team ball sport players: A systematic review" submitted to Sports Medicine - Open.

Authors: Jessica B. Farley[1], Lily M. Barrett[2], Justin W. L. Keogh[1,3,4,5], Carl T. Woods[6], Nikki Milne[1]

[1] Faculty of Health Sciences and Medicine, Bond Institute of Health and Sport, Bond University, Gold Coast, Australia
[2] Physical Education and Sport Sciences, University of Limerick, Limerick, Ireland
[3] Sports Performance Research Centre New Zealand, AUT University, Auckland, New Zealand
[4] Cluster for Health Improvement, Faculty of Science, Health, Education and Engineering, University of the Sunshine Coast, Sunshine Coast, Australia
[5] Kasturba Medical College, Mangalore, Manipal Academy of Higher Education, Manipal, Karnataka, India
[6] Institute for Health and Sport, Victoria University, Melbourne, Australia

Corresponding author: Jessica B. Farley, Email: jfarley@bond.edu.au

1. female/
2. female*.af
3. women/
4. wom#n.af
5. girl*.af
6. 1 OR 2 OR 3 OR 4 OR 5
7. athletes/
8. athlet*.af
9. player*.af
10. 7 OR 8 OR 9
11. 6 AND 10
12. ball sport*.af
13. ball game*.af
14. ballgame*.af
15. basketball/
16. basketball.af
17. football/
18. football.af
19. soccer/
20. soccer.af
21. rugby.af
22. volleyball/
23. volleyball.af
24. cricket.af
25. baseball/
26. baseball.af
27. softball.af
28. handball.af
29. netball.af
30. oztag.af
31. futsal.af
32. 12 OR 13 OR 14 OR 15 OR 16 OR 17 OR 18 OR 19 OR 20 OR 21 OR 22 OR 23 OR 24 OR 25 OR 26 OR 27 OR 28 OR 29 OR 30 OR 31
33. 11 AND 32
34. physical fitness/

35. fitness.af
36. physical endurance/
37. endurance.af
38. cardiovascular.af
39. cardiorespiratory.af
40. aerobic.af
41. anaerobic threshold/
42. anaerobic.af
43. ventilatory threshold.af
44. ventilator thresholds.af
45. oxygen consumption/
46. oxygen consumption.af
47. muscle strength/
48. strength.af
49. force.af
50. power.af
51. anthropometry/
52. anthropometr*.af
53. body height/
54. height.af
55. body weight/
56. weight.af
57. body mass index/
58. mass.af
59. body weights measures/
60. body composition/
61. composition.af
62. body size.af
63. body fat.af
64. muscle.af
65. bone density/
66. bone.af
67. musculoskeletal development/
68. development.af
69. growth.af
70. flexibility.af
71. extensibility.af
72. muscle length.af
73. range of motion articular/
74. range of motion.af
75. joint range.af
76. joint mobility.af
77. joint hypomobility.af
78. joint stiffness.af
79. joint hypermobility.af
80. joint instability/
81. laxity.af
82. psychomotor performance/
83. psychomotor performance.af
84. motor performance.af
85. motor ability.af
86. motor function.af
87. motor skills/
88. motor skill*.af
89. coordination.af
90. motor competenc*.af

91. motor proficienc*.af
92. motor development.af
93. motor activity/
94. motor activity.af
95. fine motor.af
96. gross motor.af
97. (fundamental adj3 movement).af
98. (fundamental adj3 skill).af
99. movement/
100. movement.af
101. muscular sense.af
102. proprioception/
103. proprioception.af
104. kinesthesis/
105. kinesthesia.af
106. dexterity.af
107. precision.af
108. postural balance/
109. balance.af
110. control.af
111. body equilibrium.af
112. speed.af
113. agility.af
114. reaction time.af
115. change of direction.af
116. run*.af
117. sprint*.af
118. jump*.af
119. throw*.af
120. catch*.af
121. strik*.af
122. kick*.af
123. profil*.af
124. characteristic*.af
125. physical.af
126. physiological.af
127. exercise test/
128. test*.af
129. measur*.af
130. evaluation.af
131. 34 OR 35 OR 36 OR 37 OR 38 OR 39 OR 40 OR 41 OR 42 OR 43 OR 44 OR 45 OR 46 OR 47 OR 48 OR 49 OR 50 OR 51 OR 52 OR 53 OR 54 OR 55 OR 56 OR 57 OR 58 OR 59 OR 60 OR 61 OR 62 OR 63 OR 64 OR 65 OR 66 OR 67 OR 68 OR 69 OR 70 OR 71 OR 72 OR 73 OR 74 OR 75 OR 76 OR 77 OR 78 OR 79 OR 80 OR 81 OR 82 OR 83 OR 84 OR 85 OR 86 OR 87 OR 88 OR 89 OR 90 OR 91 OR 92 OR 93 OR 94 OR 95 OR 96 OR 97 OR 98 OR 99 OR 100 OR 101 OR 102 OR 103 OR 104 OR 105 OR 106 OR 107 OR 108 OR 109 OR 110 OR 111 OR 112 OR 113 OR 114 OR 115 OR 116 OR 117 OR 118 OR 119 OR 120 OR 121 OR 122 OR 123 OR 124 OR 125 OR 126 OR 127 OR 128 OR 129 OR 130
132. 33 AND 131
133. risk/
134. risk factors/
135. risk*.af
136. factor*.af
137. causality/
138. caus*.af
139. predict*.af

140. predispos*.af
141. 133 OR 134 OR 135 OR 136 OR 137 OR 138 OR 139 OR 140
142. exp "Wounds and injuries"/
143. exp "athletic injuries"/
144. injur*.af
145. 142 OR 143 OR 144
146. 141 AND 145
147. 132 AND 146
148. *Limit to English language*
