# Supplementary material for: The relationship between physical fitness attributes and sports injury in female, team ball sport players: a systematic review
Source: Sports Med Open. 2020 Sep 14;6:45. doi: 10.1186/s40798-020-00264-9 (PMC7490320; doi:10.1186/s40798-020-00264-9)
Supplement: Supplementary file 2 — Additional file 2. Level of certainty decision-making flow chart. [file 40798_2020_264_MOESM2_ESM.pdf]

Online Resource 2

Flow chart of the decision-making process for levels of certainty, based on the level of evidence of studies contributing to the summary conclusion (adapted from: van Sluijs EM, McMinn AM, Griffin SJ. Effectiveness of interventions to promote physical activity in children and adolescents: Systematic review of controlled trials. Bmj. 2007; 335(7622): 703. doi:10.1136/bmj.39320.843947.BE).

Title: The relationship between physical fitness attributes and sports injury in female, team ball sport players: A systematic review

Journal: Sports Medicine - Open

Authors: Jessica B. Farley[1], Lily M. Barrett[2], Justin W. L. Keogh[1,3,4,5], Carl T. Woods[6], Nikki Milne[1]

[1] Faculty of Health Sciences and Medicine, Bond Institute of Health and Sport, Bond University, Gold Coast, Australia
[2] Physical Education and Sport Sciences, University of Limerick, Limerick, Ireland
[3] Sports Performance Research Centre New Zealand, AUT University, Auckland, New Zealand
[4] Cluster for Health Improvement, Faculty of Science, Health, Education and Engineering, University of the Sunshine Coast, Sunshine Coast, Australia
[5] Kasturba Medical College, Mangalore, Manipal Academy of Higher Education, Manipal, Karnataka, India
[6] Institute for Health and Sport, Victoria University, Melbourne, Australia

Corresponding author: Jessica B. Farley, Email: jfarley@bond.edu.au

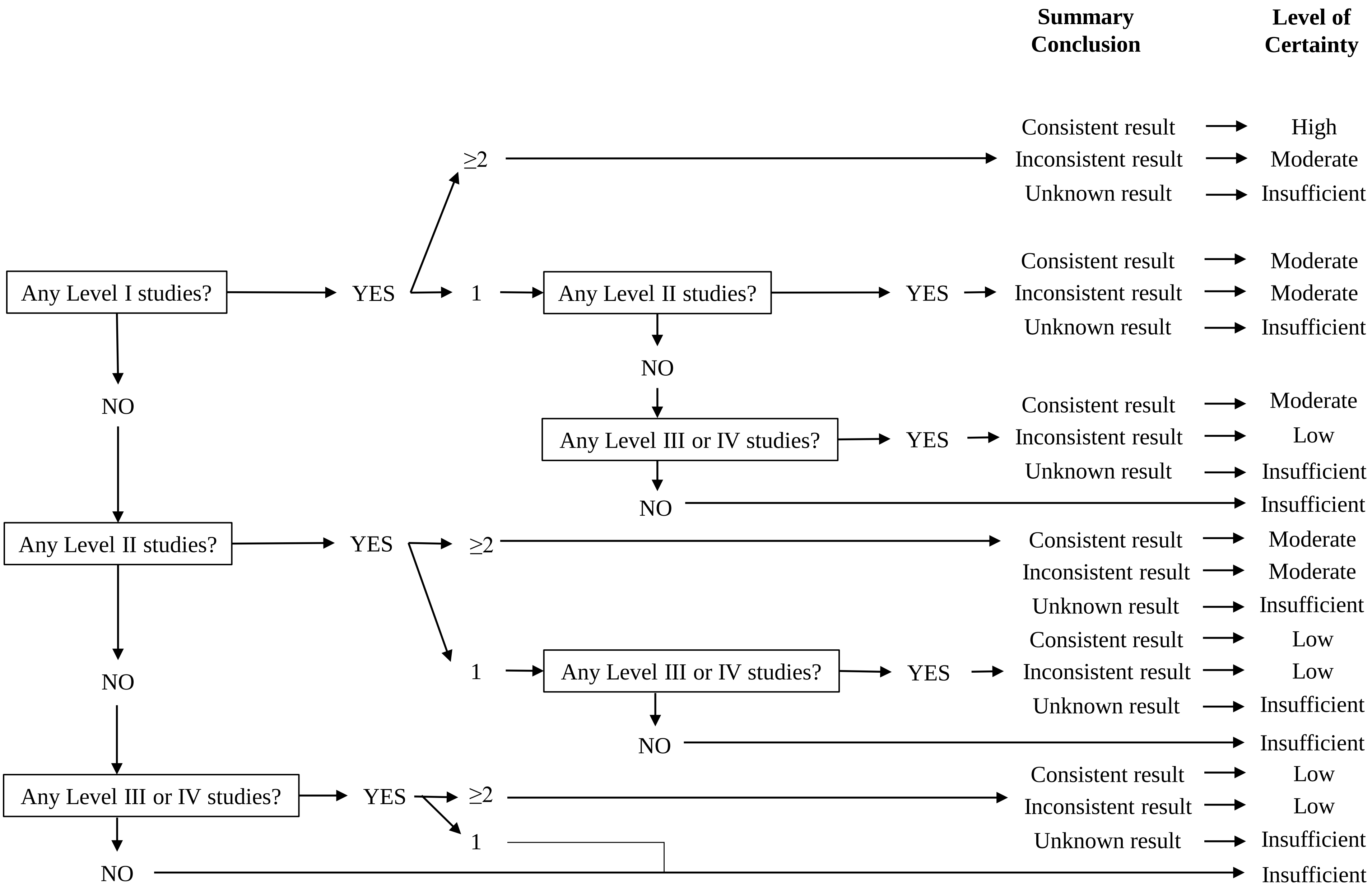

NOTE: Consistent result includes 'clear association' or 'no association' summary conclusions. Inconsistent result includes 'inconsistent association' summary conclusion. Unknown result includes 'unknown' summary conclusion.
