# Supplementary material for: The relationship between physical fitness attributes and sports injury in female, team ball sport players: a systematic review
Source: Sports Med Open. 2020 Sep 14;6:45. doi: 10.1186/s40798-020-00264-9 (PMC7490320; doi:10.1186/s40798-020-00264-9)
Supplement: Supplementary file 3 — Additional file 3. Data extraction table including characteristics of studies included in the review. [file 40798_2020_264_MOESM3_ESM.pdf]

Online Resource 3

Data extraction table including characteristics of studies included in the review titled, “The relationship between physical fitness attributes and sports injury in female, team ball sport players: A systematic review” submitted to Sports Medicine - Open.

Authors: Jessica B. Farley[1], Lily M. Barrett[2], Justin W. L. Keogh[1,3,4,5], Carl T. Woods[6], Nikki Milne[1]

[1] Faculty of Health Sciences and Medicine, Bond Institute of Health and Sport, Bond University, Gold Coast, Australia
[2] Physical Education and Sport Sciences, University of Limerick, Limerick, Ireland
[3] Sports Performance Research Centre New Zealand, AUT University, Auckland, New Zealand
[4] Cluster for Health Improvement, Faculty of Science, Health, Education and Engineering, University of the Sunshine Coast, Sunshine Coast, Australia
[5] Kasturba Medical College, Mangalore, Manipal Academy of Higher Education, Manipal, Karnataka, India
[6] Institute for Health and Sport, Victoria University, Melbourne, Australia

Corresponding author: Jessica B. Farley, Email: jfarley@bond.edu.au

Study Author (Year)	Female Participants Analysed	Study Design (Level of evidence)	Physical Fitness Measures Investigated	Sports Injury Outcome (Definition category)	Relationship Findings	
					Univariate analyses	Multivariate analyses
**HANDBALL**						
Achenbach et al. (2019) [95]	Elite junior (n = 68)	Prospective cohort study (Level II)	Shoulder strength (isometric IR & ER, eccentric ER, strength ratios), shoulder ER & IR ROM (ER gain, glenohumeral IR deficit, total ROM arc gain)	Overuse shoulder injuries (All-complaints)	Logistic regression analysis revealed risk factors for overuse shoulder injury included higher than normal differences (>7.5º) between dominant and non-dominant shoulder ER gain (OR 15.20, 95% CI 1.1-185.3, p = 0.025) and glenohumeral IR deficit (OR 12.50, 95% CI 1.4-114.6, p = 0.014). Relative eccentric shoulder ER strength <2.90 N/kg was not associated with risk of overuse shoulder injury (OR 1.38, 95% CI 0.1-13.3, p-value not published). *NOTE: Relationship results between remaining measures and injury were not published separately for females.*	Not performed.
Edouard et al. (2013) [96]	Elite junior (n = 16)	Prospective cohort study (Level II)	Shoulder ER & IR strength (dominant side deficit, strength ratios)	Shoulder injuries (Time-loss)	Imbalanced concentric ER:IR (<0.69) at 240º/s and eccentric IR:concentric ER (>1.61) at 60º/s shoulder strength ratios were associated with shoulder injury (RR 2.08-2.57, $p < 0.05$). All concentric and eccentric dominant side deficits, concentric ratios at 60º/s and 120º/s, and eccentric ER:concentric IR ratio at 60º/s were not significantly associated with shoulder injury (RR 1.24-1.88, $p > 0.05$). Logistic regression analysis also concluded a player who presented with at least two imbalanced muscular strength profiles was 2.57 times more likely to suffer a shoulder injury (RR 2.57, 95% CI 1.60-3.54, $p < 0.05$).	Not performed.

**BASKETBALL**						
Barber Foss et al. (2012) [85]	Non-elite junior (n = 262*) **athlete seasons*	Prospective cohort study (Level II)	BMI, BMI z-score, body fat percentage	Patellofemoral pain (Time-loss)	Bivariate correlation analysis showed no significant associations between relative body fat percentage (r = 0.03, p-value not published), BMI (r = 0.06, p-value not published), or BMI z-score (r = 0.06, p-value not published) to patellofemoral pain incidence status. *NOTE: Bivariate correlation between body composition measures and severity of symptoms of patellofemoral pain were not published.*	Multivariate logistic regression model including combined body composition measures did not significantly predict incidence (R^2 = 0.006-0.011, p-value not published) or severity of symptoms (adjusted R^2 = 0.04, $p > 0.05$) in youth female basketballers with patellofemoral pain.
Kofotolis & Kellis (2007) [86]	Elite senior (n = 204)	Prospective cohort study (Level II)	Height, weight	Ankle sprain (Time-loss)	Not performed.	In the presence of history of previous ankle sprain, age, training experience, and no use of external ankle support, a backward, stepwise, multivariate logistic regression analysis concluded body mass (OR 1.002, 95% CI 0.905-1.113, $p > 0.05$) and height (OR 0.952, 95% CI 0.683-1.099, $p > 0.05$) were not significant predictors of ankle sprain.
Payne et al. (1997) [93]	Non-elite senior (n = 31)	Prospective cohort study (Level II)	Ankle INV, EVR, DF, & PF strength, ankle DF flexibility	Ankle injury (Medical attention & Time-loss)	Not performed.	Stepwise multiple regression analysis concluded ankle joint strength and flexibility were not significant predictors of ankle injury with ankle proprioception measures included in the model. *NOTE: Relationship data and p-values not published.*
Plisky et al. (2006) [87]	Non-elite junior (n = 105)	Prospective cohort study (Level I)	SEBT	LE injury (Time-loss)	Univariate analysis revealed anterior right/left reach distance difference of ≥4 cm and decreased normalised anterior, posteromedial, posterolateral, and composite reach distances bilaterally were significantly associated with LE injury (OR 2.3-5.7, $p < 0.05$).	After adjusted for gender, grade, previous injury, participation in a neuromuscular training program since initial measurement, LE tape/brace use, and all potential factors found to be associated with risk of LE injury, multivariate analysis concluded the only significant predictor of LE injury was normalised composite right reach distance ≤94% of limb length (OR 6.5, 95% CI 2.4-17.5, $p < 0.05$). *NOTE: All other physical fitness variables investigated were deemed not significant.*

Shimozaki et al. (2018) [88]	Non-elite junior (n = 168)	Prospective cohort study (Level II)	BMI, knee FLEX & EXT strength, hip ABD strength, generalised joint laxity (Beighton score), postural sway	Non-contact ACL injury (Medical attention)	Not performed.	Greater BMI (OR 1.466, 95% CI 1.101-1.952, p = 0.009) and hip ABD muscle strength (OR 20.797, 95% CI 1.35-320.354, p = 0.03) were detected by multivariate logistic regression as independent risk factors for non-contact ACL injuries. Other predictor variables included in the model that were not significant risk factors were generalised joint laxity (OR 0.751, 95% CI 0.522-1.081, p-value not published), knee FLEX strength (OR 1.982, 95% CI 0.024-164.405, p-value not published), knee EXT strength (OR 1.476, 95% CI 0.107-20.453, p-value not published), and postural sway (OR 5.694, 95% CI 1.021-31.739, p-value not published).
Yentes et al. (2014) [92]	Collegiate (n = 11)	Prospective cohort study (Level II)	Ankle INV & EVR strength (difference between barefoot and shod conditions, difference in time to peak torque, strength ratios)	LE injury (Time-loss)	Spearman rho correlation demonstrated a significant relationship between a large difference in barefoot and shod conditions peak EVR torque and LE injuries (ρ = 0.78, p = 0.02). No other strength variables demonstrated a significant relationship with LE injury (ρ = 0.06-0.32, p = 0.44-0.89).	Not performed.
**NETBALL**						
Attenborough et al. (2017) [97]	Elite and non-elite senior (n = 94)	Prospective cohort study (Level II)	Height, weight, ankle INV-EVR PROM, demi-pointe test, foot lift test, SEBT, vertical jump	Ankle sprain (Time-loss)	A preseason posterior-medial reach distance ≤77.5% of leg length on the SEBT was identified as a predictive risk factor for the development of an ankle sprain by univariate analysis (OR 4.04, 95% CI 1.00-16.35, p = 0.04). All other physical fitness variables were deemed not significantly associated with ankle sprain (OR 1.47-3.78, p = 0.05-0.41).	Not performed.

Hopper et al. (1995) [98]	Elite and non-elite senior (n = 72)	Prospective cohort study (Level II)	Height, weight, somatotype, alactic power test, generalised joint laxity (Beighton score), single leg stance, vertical jump	LE or back injury (Medical attention)	Univariate logistic regression demonstrated an increased risk of LE or back injury with greater jumping ability ($p < 0.005$), superior anerobic fitness ($p < 0.05$), and lower endomorphy component ($p < 0.05$). *NOTE: Relationship data not published. All other physical fitness variables investigated were deemed not significant.*	Multiple logistic regression concluded vertical jump (with arms) was a predictive measure of injury, however anaerobic fitness was no longer a significant risk factor. A low score on the endomorphy scale remained a significant risk factor. *NOTE: Relationship data and p-values not published. Relationship results between remaining physical fitness variables and injury were not published.*
Hopper (1997) [99]	Elite senior and junior (n = 213)	Cross-sectional study (Level III)	Somatotype	LE or back injury (Medical attention)	There were no relationships between the incidence of LE or back injuries and endomorphic ($F_{2,195}$ = 0.53, p = 0.46), mesomorphic ($F_{2,195}$ = 0.23, p = 0.63), and ectomorphic ($F_{2,195}$ = 1.89, p = 0.16) somatotype scales.	Not performed.
Smith et al. (2005) [100]	Non-elite junior (n = 200)	Cross-sectional study (Level III)	Generalised joint laxity (Beighton score)	All injuries (Time-loss)	Not performed.	Logistic regression analysis showed increased generalised joint laxity to be an independent risk factor for netball injury (OR 2.998-3.364, p = 0.01-0.015) when adjusted for age, years playing netball, games played a week, and court positions.
**RUGBY**						
Armstrong & Greig (2018) [104]	Collegiate (n = 64)	Prospective cohort study (Level II)	FMS™	All injuries (total days injured) (Time-loss)	Linear regression analysis revealed FMS™ composite score was a significant predictor of total days injured for all injuries (r^2 = 0.07, F = 4.71, p = 0.03) and when analysed with contusions removed (r^2 = 0.07, F = 4.06, p = 0.04). The best independent predictor for injury was left in-line lunge for all injuries and with contusions removed (r^2 = 0.12-0.13, F = 7.87-8.23, p = 0.01). *NOTE: All other physical fitness measures investigated were deemed not significant.*	Multiple linear regression analysis of all FMS™ components combined together revealed a significant predictor of total days injured for all injuries (r^2 = 0.37, F = 2.54, p = 0.01) and with contusions removed (r^2 = 0.39, F = 2.26, p = 0.03). Stepwise multiple hierarchical linear regression showed six components of the FMS™ (bilateral in-line lunge, left rotary stability, bilateral hurdle step, right shoulder mobility) significantly predicted 19% of variance in total days injured for all injuries (R^2 = 0.19, F = 4.81, p = 0.01) and with contusions removed (R^2 = 0.19, F = 4.83, p = 0.01). *NOTE: All other physical fitness measures investigated were deemed not significant.*

**SOCCER**						
Blokland et al. (2017) [62]	Elite senior (n = 114)	Prospective cohort study (Level II)	Generalised joint laxity (Beighton score and individual components)	All injuries, thigh injury, knee injury, ankle injury, all non-contact injuries, all recurrent injuries, all match injuries (Time-loss)	Not performed.	When adjusted for age, BMI, and soccer exposure, Poisson and logistic regression analyses concluded increased generalised joint laxity is not a risk factor for all musculoskeletal injuries, thigh, knee, and ankle injuries separately, and any non-contact, recurrent, or match injury categories (IRR 0.64-1.74, p = 0.219-0.927; OR 0.57-2.61, p = 0.138-0.904). None of the individual items of the Beighton score significantly impacted all musculoskeletal injury risk (IRR 0.73-1.25, p = 0.176-0.569; OR 0.38-2.59, p = 0.078-0.998). Knee hyperextension ROM had no significant effect on the incidence of knee injury (IRR 1.15, 95% CI 0.58-2.27, p = 0.695; OR 0.89, 95% CI 0.32-2.45, p = 0.816). *NOTE: Relationship data between remaining individual Beighton components and injury categories were not published.*
Cheng et al. (2019) [63]	Elite junior (n = 88)	Prospective cohort study (Level II)	BMI, hip IR ROM	Lower body injury, lumbopelvic injury, hip injury, knee injury, overuse lower body injury, incomplete recovery from lower body injury, multiple lower body injuries (Time-loss)	Not performed.	In the presence of age at baseline testing, age when started playing soccer, BMI, and at least 1 provocative hip screening test, hip IR ROM ≤20º is not predictive of lower body, lumbopelvic, hip, knee, overuse, incomplete recovery, or multiple injuries concluded by a stepwise multivariable logistic regression (p > 0.05). *NOTE: Relationship data not published. Relationship results for the association of BMI and all injury categories were not published.*
Emery et al. (2005) [67]	Non-elite junior (n = 164)	Prospective cohort study (Level II)	BMI, 20-m shuttle run (VO_2 max), dynamic balance (eyes closed), vertical jump	All injuries (Medical attention & Time-loss)	There was no increased sports injury risk associated with suboptimal VO_2 max (RR 0.85, 95% CI 0.35-1.98, p = 0.69) or decreased vertical jump (RR 1.03, 95% CI 0.46-2.3, p = 0.82). *NOTE: Relationship results between the remaining variables and injury were not published separately for females.*	Not performed.

Emery & Meeuwisse (2006) [66]	Non-elite junior (n = 164 outdoor, 75 indoor)	Prospective cohort study (Level II)	BMI, 20-m shuttle run (VO_2 max), dynamic balance (eyes closed), vertical jump	All injuries (Medical attention & Time-loss)	There was no apparent increased sports injury risk associated with suboptimal VO_2 max (RR 0.85, 95% CI 0.35-1.98, *p* = 0.69) or decreased vertical jump (RR 1.03, 95% CI 0.46-2.3, *p* = 0.82) in female, junior outdoor soccer players. Similar results were found in female, junior indoor soccer players (RR 1.67-2.57, *p* = 0.11-0.41). *NOTE: Relationship results between the remaining variables and injury were not published separately for females. Results for outdoor soccer players were not included in the data synthesis as duplicate data [see Emery et al. (2005)].*	Not performed.
Faude et al. (2006) [68]	Elite senior (n = 143)	Prospective cohort study (Level II)	Height, weight, BMI	All injuries (Time-loss)	Players >175 cm showed a significant increased injury risk compared to those of intermediate height (OR 9.64, 95% CI 1.56-58.52, *p* = 0.01). The shortest players did not demonstrate a significant increased injury risk (OR 1.70, 95% CI 0.59-4.85, *p* =0.34). Weight and BMI were not significantly associated with increased injury risk (OR 0.56-1.92, *p* = 0.27-0.44).	Not performed.
Hägglund & Waldén (2016) [69]	Non-elite junior (n = 4556)	Prospective cohort study (Level II)	BMI	Acute knee injury, ACL injury (Time-loss)	Cox regression analysis concluded a greater BMI (>19.9 kg/m^2) was significantly associated with increased risk for acute knee injury (HR 1.53, 95% CI 1.00-2.32, *p* = 0.048; Sensitivity 55%, Specificity 56%), however not with ACL injury (HR 2.40, 95% CI 0.96-6.00, *p* = 0.063).	Multivariate Cox regression analysis revealed BMI was not associated with increased rate of acute knee injury (HR 1.26, 95% CI 0.80-1.97, *p* > 0.05) in the presence of age >14 years, achieved menarche, previous acute knee injury, presence of current knee complaints, familial disposition to ACL injury, and exposure to artificial turf playing surfaces. BMI was also not associated with ACL injury (HR 1.56, 95% CI 0.61-3.99, *p* > 0.05) in the presence of age >14 years, born in the first half of the year, achieved menarche, and familial disposition to ACL injury.

Koenig & Puckree (2015) [70]	Non-elite junior (n = 68)	Cross-sectional study (Level III)	BMI, Modified Clinical Test for Sensory Integration and Balance	All injuries (Not published)	Pearson's correlations revealed there were no significant associations between injury and BMI ($r = 0.056$, $p = 0.62$) or static balance ($r = -0.072$ to 0.073, $p = 0.52-0.98$).	Not performed.
McCann et al. (2018) [72]	Collegiate (n = 43)	Prospective cohort study (Level II)	Height, weight, BMI	Lateral ankle sprains (Medical attention & Time-loss)	Binary logistic regression revealed height was a significant predictor of lateral ankle sprain (OR 1.30, 95% CI 1.00-1.70, $p = 0.05$). ROC curve analysis further demonstrated moderate predictive utility of height (AUC = 0.73 [0.58, 0.89]; $p = 0.04$). Specifically, players ≥167.6 cm in height had 7.5 times greater odds of sustaining a lateral ankle sprain than those of shorter height. Weight (OR 1.00, 95% CI 0.84-1.19, $p = 0.95$) and BMI (OR 0.74, 95% CI 0.46-1.20, $p = 0.22$) were not significantly associated with lateral ankle sprain.	Not performed.
Ness et al. (2017) [74]	Collegiate (n = 71)	Retrospective cohort study (Level II)	Height, weight, Gauntlet test	LE injuries (Time-loss)	Logistic regression concluded number of Gauntlet trials to success was significantly predictive of time-loss LE injury (OR 3.578, $p < 0.02$); however, height (OR 0.068, *p*-value not published) and weight (OR 0.996, *p*-value not published) were not significant predictors.	Only the number of Gauntlet trials to success was significantly predictive of time-loss LE injury by multivariate analysis (OR 4.508, $p < 0.02$) in the presence of age, weight (OR 1.015, *p*-value not published), and height (OR 0.027, *p*-value not published). ROC curve analysis results indicated the Gauntlet test had good diagnostic properties for subsequent injury (AUC = 0.714, Youden index was two Gauntlet test trials for success, Sensitivity 0.92, Specificity 0.46).

Nilstad et al. (2014) [75]	Elite senior (n = 173)	Prospective cohort study (Level II)	Height, weight, BMI, isokinetic maximal knee EXT & FLEX strength, maximal hip ABD strength, 1-RM leg press, generalised joint laxity (Beighton score), SEBT	LE injury, thigh injury, knee injury, ankle injury, leg/foot injury (Time-loss)	Univariate analyses revealed heavier weight (OR 1.34, 95% CI 1.07-1.68, p = 0.01) and BMI (OR 1.55, 95% CI 1.24-1.94, $p \leq 0.001$) were risk factors for LE injuries. Greater 1-RM leg press was significantly associated with ankle injury (OR 1.47, 95% CI 1.05-2.05, p = 0.02). No other physical fitness variables were significantly associated with LE, thigh, knee, or lower leg/foot injuries, independently by univariate analysis (OR 0.70-1.44, p = 0.09-0.96). *NOTE: Relationship results between height and weight and thigh, knee, ankle, and lower leg/foot injuries were not published.*	Multivariate analysis concluded BMI to be a significant predictor of LE injury (OR 1.51, 95% CI 1.21-1.90, p = 0.001) in the presence of age, previous ACL injury, knee valgus angles, and foot pronation. BMI was also a significant predictor of thigh injury (OR 1.51, 95% CI 1.08-2.11, p = 0.01) when hamstring strength (OR 1.45, 95% CI 0.98-2.16, p = 0.06) and previous hamstring injury included in the model. BMI was not predictive of lower leg/foot injuries (OR 1.40, 95% CI 0.90-2.17, p = 0.14) in the presence of previous knee injury and age. 1-RM leg press was not a significant predictor of ankle injuries (OR 1.41, 95% CI 0.97-2.06, p = 0.07) in the presence of knee valgus angle, foot pronation, and age. *NOTE: Physical fitness variables were not explored in the multivariate analysis for knee injuries. Relationship results between remaining physical fitness variables and injury categories were not published.*
Niyonsenga & Phillips (2013) [76]	Elite senior (n = 300)	Cross-sectional study (Level III)	BMI, knee EXT and ankle DF ROM	All injuries (Three-season history) (Time-loss)	Chi-square analysis revealed excessive ankle DF (p = 0.006) and knee EXT (p = 0.037) ROM to be significantly associated with three-season history of injury. BMI was not significantly associated with three-season history of injury (p = 0.105). *NOTE: Relationship data not published.*	Not performed.

O'Kane et al. (2017) [77]	Elite junior (n = 351)	Prospective cohort study (Level II)	Height, weight, hip FLEX, EXT, ABD, ADD, & ER strength, concentric knee EXT & FLEX strength, generalised joint laxity (Beighton score)	Overuse LE injury, overuse knee injury (Time-loss)	Poisson regression analysis revealed a 1 SD increase in hamstring (RR 0.65, 95% CI 0.46-0.91), quadriceps (RR 0.70, 95% CI 0.50-0.98), hip FLEX (RR 0.72, 95% CI 0.51-1.00), and hip ER strength (RR 0.65, 95% CI 0.46-0.91) were each significantly associated with a decreased risk in overuse knee injury. Hip EXT, ABD, and ADD strength were not statistically associated with overuse knee injury (RR 0.79-1.06). No statistically significant associations existed between all muscle strength measures and overuse LE injury (RR 0.97-1.13). Joint hypermobility was also not associated with risk of overuse LE (RR 0.66, 95% CI 0.31-1.39) or knee (RR 0.68, 95% CI 0.23-2.02) injury. *NOTE: p-values not published. Relationship results between height and weight and overuse LE and knee injuries were not published.*	Not performed.
Östenberg & Roos (2000) [78]	Elite and non-elite senior (n = 123)	Prospective cohort study (Level I)	Height, weight, BMI, multi-stage fitness test, concentric knee FLEX & EXT strength, generalised joint laxity (Beighton score), vertical jump, single leg hop for distance, square hop test	All injuries, knee injuries (Time-loss)	*NOTE: Univariate logistic regression analyses between potential physical fitness predictors and injuries were performed, however results were not published.*	Significant risk factors for any injury were an increased generalised joint laxity (OR 5.3, 95% CI 2.0-13.5, *p* < 0.001) and a high performance in the functional square-hop test (OR 4.3, 95% CI 1.7-10.5, *p* = 0.002) when in the presence of each other and age. Generalised joint laxity was also shown to be a significant risk factor for knee injury (OR 5.0, 95% CI 1.3-18.9, *p*-value not published). *NOTE: Other predictors included in the model for knee injury not published. Relationship results between remaining measures and injury were not published.*

Räisänen et al. (2018) [79]	Elite junior (n = 113)	Prospective cohort study (Level I)	Height, weight, BMI, single leg squat	Acute LE injuries, acute non-contact LE injuries (Medical attention & Time-loss)	Height, weight, BMI, and inability to perform a valid single-leg squat were not associated with acute LE (OR 1.01-1.50, p = 0.40-0.76) or acute non-contact LE injury (OR 0.94-3.09, p = 0.12-0.64) by univariate analysis.	Multivariate analysis concluded that when weight and inability to perform a valid single leg squat were included together in a model, they both were not associated with non-contact LE injuries (OR 1.43-2.93, p = 0.07-0.52). *NOTE: Height and BMI were not investigated by multivariate analysis for non-contact LE injuries. All physical fitness variables were also not investigated by multivariate analysis for relationship with acute LE injury for females only.*
Söderman et al. (2001) [80]	Non-elite junior (n = 146)	Prospective cohort study (Level II)	Knee EXT & FLEX strength (H:Q ratio, thigh torque side-to-side difference), generalised joint laxity (Beighton score), ankle DF ROM, hamstring extensibility, postural sway	Traumatic LE injuries Overuse LE injuries (Time-loss)	A lower concentric H:Q ratio (OR 0.95, 95% CI 0.89-0.999, p = 0.046), excessive generalised joint laxity (OR 3.10, 95% CI 1.19-8.01, p = 0.02), and low postural sway (OR 0.46, 95% CI 0.22-0.96, p = 0.04) were found to be significant risk factors for traumatic LE injuries. Concerning overuse LE injuries, a higher H:Q ratio (OR 1.13, p = 0.004) and side-to-side differences in hamstring extensibility (OR 3.36, p = 0.049) and ankle DF (OR 7.06, p = 0.02) were independently related to risk of injury. *NOTE: All other physical fitness variables investigated were deemed not significant.*	When the effect of all variables with p < 0.20 were analysed in a multivariate logistic regression, a low postural sway (OR 0.31, p = 0.005) and a lower H:Q ratio (OR 0.93, p = 0.02) were found to significantly increase the risk of traumatic leg injury (other significant predictor variables included in the model were knee hyperextension alignment and soccer exposure). *NOTE: Relationship results between remaining measures and injury were not published. Multivariate analysis between physical fitness measures and overuse LE injury was not investigated.*
Sugimoto et al. (2018) [82]	Non-elite junior (n = 160)	Cross-sectional study (Level III)	Weight, BMI	History of musculoskeletal injuries (Medical attention)	Not performed.	Players who were heavier in weight (β = -0.097; adjusted OR 0.908, 95% CI 0.834-0.989, p = 0.026) and had a higher BMI (β = 0.358; adjusted OR 1.430, 95% CI 1.074-1.904, p = 0.014) were independently associated with a history of musculoskeletal injuries determined by binary logistic regression in the presence of age, hours of training per week, season participated, and status of playing positions.

Watson et al. (2017) [84]	Non-elite junior (n = 54)	Case-control study (Level III)	BMI, maximal cycle exercise test (VO_2 max, time to exhaustion)	All injuries (Time-loss)	Poisson regression analysis revealed a higher preseason VO_2 max was a significantly associated with a decreased risk of injury sustained during the season (OR 0.95, 95% CI 0.90-0.99, $p = 0.046$). No significant relationships were identified between injury and BMI (OR 1.02, 95% CI 0.87-1.19, $p = 0.80$) or max time to exhaustion on a cycle exercise test (OR 0.89, 95% CI 0.75-1.06, $p = 0.18$).	Not performed.
**SOCCER AND BASKETBALL**						
Myer et al. (2008) [73]	Non-elite junior (high school) and collegiate (n = 95)	Case-control study (Level III)	Generalised joint laxity (individual components)	ACL injury (Medical attention)	Binary logistic regression concluded no association between individual generalised joint laxity measures and ACL injury (OR 1.06-2.34, $p = 0.11$-0.92).	Multivariate logistic regression revealed a positive measure of knee hyperextension increased the odds of ACL injury 5-fold (OR 4.78, 95% CI 1.24-18.44; $p = 0.02$). Predictor variables included in the model: generalised joint laxity measures taken at the fifth metacarpal and thumb, side-to-side differences in anterior-posterior tibiofemoral translation, and previous knee injury.
**SOCCER AND HANDBALL**						
Steffen et al. (2016) [81]	Elite senior (n = 867)	Prospective cohort study (Level II)	Quadriceps, hamstring, and hip ABD strength, leg press, H:Q ratio	Non-contact ACL injury (Medical attention)	Not performed.	LE strength (OR 0.84-1.23, $p > 0.05$) was not associated with an increased risk of non-contact ACL injury concluded by multivariate logistic regression (adjusted for the effect of sport, dominant leg, any previous ACL injury, height, and isokinetic dynamometer used).

**SOCCER, BASKETBALL, VOLLEYBALL**						
Chorba et al. (2010) [64]	Collegiate (n = 38)	Prospective cohort study (Level II)	FMS^{TM}	All injuries (Medical attention)	Linear regression analysis revealed a significant correlation between composite FMS^{TM} score and risk of injury in players without a previous history of ACL injury (r = -0.8214, r^2 = 0.6748, p = 0.0450). FMS^{TM} score ≤14 resulted in a 4.6 increase risk of injury (OR 4.583, 95% CI 0.994-21.127; sensitivity 0.647, specificity 0.714). No significant correlation was found when all subjects were considered (r^2 = 0.5892, p = 0.0748).	Not performed.
Landis et al. (2018) [71]	Collegiate (n = 187)	Prospective cohort study (Level II)	Body mass, tibia length, H:Q ratio, FMS^{TM}	Non-contact ACL injury, non-contact LE injury (Medical attention &Time-loss)	Exact logistic regression model indicated that FMS^{TM} composite score was a significant predictor of non-contact ACL injury (OR 0.40, 95% 0.17-0.93, p = 0.03), however not associated with non-contact LE injury (OR 0.64, 95% CI 0.401-1.03, p = 0.06).	When controlled for pain reported during the FMS^{TM} and prior ACL injury and/or knee surgery, the FMS^{TM} composite score was not associated with non-contact LE injury (OR 0.75, 95% CI 0.42-1.37, p = 0.35). When considering all combinations of the FMS^{TM} components in the presence of knee ABD moment clinical measures during a drop-jump landing task, logistic regression revealed no statistically significant predictors of non-contact ACL or LE injury (p > 0.05). *NOTE: Adjusted result for relationship between FMS^{TM} composite score and non-contact ACL injury not investigated.*
Warren et al. (2019) [83]	Collegiate (n = 68)	Prospective cohort study (Level II)	Hip EXT, ABD, and ER strength, single leg hop for distance, triple hop for distance, and crossover hop for distance	Non-contact lower body injury (LE or back) (Medical attention)	Logistic regression concluded greater triple hop for distance asymmetry (>12 cm) (OR 7.31, 95% CI 1.95-27.34) and weak hip ER strength (OR 6.00, 95% CI 1.11-32.54) were significantly predictive for non-contact lower body injuries. No significant associations were found between non-contact lower body injury and the remaining hop tests (OR 2.44-2.67), hip ABD strength (OR 0.43-1.75), or hip EXT strength (OR 1.28-1.43). *NOTE: p-values not published.*	Similar significant results (triple hop: OR 6.5, 95% CI 1.69-25.04, p = 0.007; hip ER strength: OR 6.09, 95% CI 1.09-34.19, p-value not published) and not significant results (single leg hop for distance: OR 1.91, 95% CI 0.52-7.02, p = 0.33; crossover hop test: OR 2.02, 95% CI 0.56-7.24, p = 0.28; hip ABD strength: OR 0.45-1.51, p = 0.26; and hip EXT strength: OR 1.03-1.10, p = 0.99) remained when adjusted for previous injury.

**SOCCER, FIELD HOCKEY, AND BASKETBALL**						
Devan et al. (2004) [65]	Collegiate (n = 53)	Prospective cohort study (Level II)	H:Q ratios (60º/s, 300º/s), IT band flexibility (Obers test)	Overuse knee injuries (Medical attention)	H:Q ratio <80% at 300º/s was associated with overuse knee injuries (p = 0.047). No associations were noted between the remaining H:Q ratios and overuse knee injuries (p > 0.05). *NOTE: Relationship data not published. Relationship result between IT band flexibility and overuse knee injury was not published.*	Not performed.
**SOCCER, FIELD HOCKEY, AND LACROSSE**						
Beynnon et al. (2001) [61]	Collegiate (n = 68)	Prospective cohort study (Level II)	Height, weight, ankle PF, DF, INV, & EVR strength, generalised joint laxity (Beighton score), ankle INV, EVR, & DF ROM, balance test	Ankle sprain (Medical attention)	Cox regression analysis revealed a significant relationship between calcaneal EVR ROM and ankle sprain (p = 0.038). All other relationships between ankle sprain and physical fitness measures were deemed not significant (p = 0.10-1.00). *NOTE: Relationship data not published.*	Not performed.
**SOFTBALL**						
Aragon et al. (2012) [101]	Collegiate (n = 65)	Cross-sectional study (Level III)	Forward/backward half-kneeling rotation test (bar in the back/front), forward/backward seated rotation test	History of shoulder or elbow injury (Time-loss)	A history of shoulder or elbow injury was associated with forward half-kneeling rotation (bar in back) (χ^2 = 4.5, p = 0.03) and seated rotation test (χ^2 = 10.3, p = 0.006). Injury prevalence was 2.75 times greater in the low (≤42.5º) relative to the high (≥47.7º) half-kneeling rotation flexibility group (χ^2 = 4.09, p = 0.043, 95% CI: 1.02, 7.32). Injury prevalence was 7.3 times (χ^2 = 3.82, p = 0.05, 95% CI: 1.00, 53.1) and 8.7 times (χ^2 = 4.65, p = 0.03, 95% CI: 1.22, 62.1) greater in the high (≥47.3º) and low (≤41.5º) seated rotation flexibility groups, respectively. Associations between a history of shoulder or elbow injury and the other flexibility measures were not statistically significant (χ^2 = 0.25-3.8, p = 0.15-0.88).	Not performed.

Hill et al. (2004) [102]	Collegiate (n = 181)	Cross-sectional study (Level III)	Height, weight	All injuries (Medical attention)	Height and weight were not significantly associated with injury incidence in collegiate softball pitchers ($p > 0.05$). *NOTE: Relationship data not published.*	Not performed.
Shanley et al. (2011) [103]	Non-elite junior (n = 103)	Prospective cohort study (Level II)	Shoulder IR, horizontal ADD, and total shoulder (ER + IR) PROM site-to-side differences	UE injury (All-complaints)	Side-to-side differences in shoulder IR, horizontal ADD, and total shoulder (ER + IR) PROM were not predictive of injury (RR 0.60-3.1, $p > 0.05$).	Not performed.
**VOLLEYBALL**						
Brumitt et al. (2019) [94]	Collegiate (n = 82)	Prospective cohort study (Level I)	Standing long jump, single leg hop for distance	Non-contact lower quarter (back or LE) injury, thigh/knee injury, ankle/foot injury (Time-loss)	Suboptimal performance in the standing long jump, single leg hop for distance, and single leg hop for distance side-to-side asymmetry individually were not associated with greater risk of non-contact lower quarter, thigh/knee, or ankle/foot injuries (RR 0.5-3.3, $p > 0.05$).	Suboptimal performance on combined tests (standing long jump <80% and bilateral single leg hop for distance <70% of one's height, and single leg hop for distance side-to-side symmetry >10%) was associated with risk of non-contact injury to the lower quarter (RR 4.6, 95% CI 2.1-10.1, $p = 0.01$) and ankle/foot injury (RR 6.3, 95% CI 2.1-19.2, $p = 0.01$), however not with non-contact thigh/knee injury (RR 2.2, 95% CI 0.3-16.1, $p > 0.05$). When side-to-side symmetry of the single leg hop test was removed, these findings were no longer significant (RR 1.9-3.3, $p > 0.05$).
**VOLLEYBALL AND BASKETBALL**						
van der Worp et al. (2012) [89]	Elite senior (n = 1218)	Cross-sectional study (Level III)	BMI, waist-to-hip ratio	Patellar tendinopathy (Medical attention)	Not performed.	BMI (OR 1.0, 95% CI 0.9-1.1, $p > 0.05$) and waist-to-hip ratio (OR 8.4, 95% CI 0.3-284.2, $p > 0.05$) were not significantly associated with patellar tendinopathy concluded by multiple logistic regression (other variables included: age, sport, sport experience, hours of training last week, training increase compared with last year, number of games last month, average hours of other sports/week, playing surface, playing level, playing position).

Walbright et al. (2017) [91]	Collegiate (n = 35)	Prospective cohort study (Level II)	FMS^{TM}, Y-balance test, single leg hop for distance	Lower quarter injury (hours lost and incidence) (Time-loss)	Components of the FMS^{TM} were not predictive of lost-time lower quarter injury occurrence (+LR 0.73-1.45, $p > 0.05$). No significant relationships were found between occurrence of lost-time lower quarter injury and Y-balance test or single leg hop for distance test (*p*-values not reported). The hurdle step component was associated with hours lost due to a lower quarter injury ($p < 0.05$). *NOTE: Relationship results not published. Relationship results between Y-balance test and single leg hop for distance test and hours lost due to lower quarter injury were not published.*	Not performed.
**VOLLEYBALL, BASKETBALL, AND HANDBALL**						
Vauhnik et al. (2008) [90]	Senior players (competition level unclear) (n = 585)	Prospective cohort study (Level II)	Height, weight, BMI, knee EXT PROM	Traumatic knee injury (Time-loss)	Logistic regression analysis revealed height, weight, BMI, and passive knee EXT were not significantly associated with traumatic knee injury (OR 1.0-1.1, $p = 0.086$-0.441).	Height was identified as a potential risk factor for traumatic knee injury in female team ball players when considered in a model with anterior tibial translation (OR 1.1, 95% CI 1.004-1.147, $p = 0.038$). *NOTE: Other physical fitness variables were not investigated.*

Abbreviations: 1-RM = 1-repetition maximum; ACL = anterior cruciate ligament; ABD = abduction; ADD = adduction; AUC = area under the curve; BMI = body mass index; CI = confidence interval; DF = dorsiflexion; ER = external rotation; EVR = eversion; EXT = extension; FLEX = flexion; FMS^{TM} = Functional Movement Screen^{TM}; HR = hazards ratio; H:Q = hamstring/quadriceps ratio; INV = inversion; IR = internal rotation; IRR = incidence rate ratio; IT band = iliotibial band; LE = lower extremity; LR = likelihood ratio; OR = odds ratio; PF = plantarflexion; PROM = passive range of motion; ROC = receiver operating characteristics; ROM = range of motion; RR = relative risk; SD = standard deviation; SEBT = star excursion balance test; UE = upper extremity; VO_2 max = maximal oxygen consumption.
